# Siloxane-tethered poly(2-ethyl-2-oxazoline) as surface modifying additives for anti-fouling silicones

**DOI:** 10.1016/j.eurpolymj.2026.114638

**Published:** 2026-03-11

**Authors:** Jenlyan Negrón Hernández, Anika S. Palacharla, Darian K. Kanu, Shane J. Stafslien, Lyndsi Vander Wal, Melissa A. Grunlan

**Affiliations:** a Department of Chemistry, Texas A&M University, College Station, Texas 77843, United States; b Department of Biomedical Engineering, Texas A&M University, College Station, Texas 77843, United States; c Department of Materials Science and Engineering, Texas A&M University, College Station, Texas 77843, United States; d Department of Coatings and Polymeric Materials, North Dakota State University, Fargo, ND 58108, United States

**Keywords:** Silicones, Antifouling, Amphiphilic, Surface modifying additives, Poly(2-ethyl-2-oxazoline)

## Abstract

While silicones are broadly utilized for medical devices, their hydrophobic nature renders them highly prone to biofouling, leading to reduced safety and efficacy. We have previously demonstrated that biofouling is reduced for silicones combined with amphiphilic surface modifying additives (SMAs) comprised of a poly(ethylene glycol) (PEG) segment and a siloxane tether (PEG-silane). However, there is a rising concern regarding the potential immunogenicity of PEG. Herein, SMAs were created wherein poly(2-ethyl-2-oxazoline) (PEtOx) was substituted for PEG. PEtOx-silane SMAs (H-Si-DMS_*m*_-*block*-PEtOx_10_; *m* = 13 and 30) were synthesized, and each incorporated into unfilled (i.e., silica-free) silicones: dimethyl silicone [MED-6019; 0% Ph], dimethyl diphenyl silicone [MED-6010; 5.8% Ph], and dimethyl diphenyl silicone [MED-6020; 8.5% Ph] at increasing concentrations (5 – 25 μmol g^−1^). Analogous PEG-silane SMAs (H-Si-DMS_*m*_-*block*-PEG_8_; *m* = 13 and 30) were likewise used to modify the silicones. The chemical crosslinking and subsequent retention of SMAs in modified silicones was confirmed. The resulting properties imparted by a particular SMA were impacted by the phenyl content of the silicone. However, PEtOx-based SMAs generally produced a greater plasticizing effect than PEG-based SMAs. Fouling against fibrinogen, a fungus, and four bacteria was assessed for silicones modified with each SMA (25 μmol g^−1^). In general, silicones modified with POx-based SMAs demonstrated greater anti-fouling behavior versus unmodified silicones. DMS_30_-POx often achieved similar anti-fouling efficacy versus PEG-based SMAs. These results point to the potential of amphiphilic POx-based SMAs to produce anti-fouling silicones.

## 1. Introduction

Silicones are widely used in the biomedical industry to construct a myriad of medical devices (e.g., catheters, shunts, facial prostheses, lenses, etc.) and coatings [1,2]. Their utility stems from unique properties that include elastomeric mechanical behavior, degradation resistance, and oxygen permeability. Such silicones are based on a siloxane polymer backbone, traditionally with dimethyl pendant groups (i.e., polydimethylsiloxane, PDMS). Phenyl silicones, having also some fraction of diphenyl pendant groups, have emerged based on excellent thermal stability, durability, and optical properties [3]. Silicones are cured via several crosslinking strategies [4–7]. For instance, moisture-cured silicones are produced via hydrolysis and condensation of functional groups (e.g., acetoxy) and are accelerated by a tin (Sn) catalyst. Addition cured silicones are formed via platinum (Pt)-catalyzed hydrosilylation reactions between silane (Si-H) and vinyl groups. Silica fillers may also be included to tailor mechanicals properties, such as increasing hardness and strength [8]. Unfortunately, because of their hydrophobic nature, silicone-based medical devices and coatings are highly susceptible to biological adhesion [1,9–11]. Adhesion is mediated by nonspecific protein adsorption wherein a conformational change orients the protein’s hydrophobic domains to the silicone surface and hydrophilic domains to the aqueous surrounding of the body [12]. The resulting accumulation of plasma proteins, bacteria, and fungi reduce device efficacy and safety by causing thrombosis, encapsulation, and infection [13–16]. Numerous strategies have been evaluated to impart antifouling behavior to silicones and other polymers, and may be passive and/or active in nature [17–23].

A surface modifying additive (SMA) is an attractive option to produce anti-fouling silicones as it can be readily incorporated via simple bulk modification (i.e., blending) prior to cure. To be effective, such SMA must afford appreciable migration of the anti-fouling species to the aqueous interface where fouling occurs. Poly(ethylene glycol) (PEG) is a hydrophilic polymer that has been extensively used in biomedical applications, and is considered the gold-standard for passive anti-fouling [24–26]. This behavior is associated with its hydration layer and steric effects that give rise to a large excluded volume that blocks underlying adsorption sites [24,27–31]. The anti-fouling behavior of PEG surfaces has been largely demonstrated on model substrates (e.g., gold [32–34], glass [35,36], and silica wafer [37–40]). In the case of PEG modification of silicones, it is imperative that PEG chains are maintained at the aqueous/biological interface at sufficient levels to effectively repel foulants. Direct surface grafting of PEG onto silicones is limited by irreversible hydrophobic recovery [41–46]. Silicones bulk modified with PEG oligomers (e.g., (EtO)_3_Si-(CH_2_)_3_-PEG_n_-OCH_3_ and CH_2_=CHCH_2_-PEG_n_-OCH_3_) are also prone to hydrophobic recovery, resulting in a lack of PEG at the aqueous interface [47–49]. We have previously reported amphiphilic SMAs comprised of a hydrophilic PEG segment and a hydrophobic dimethyl siloxane tether [(EtO)_3_Si-(CH_2_)_2_-(DMS)_*m*_-PEG_8_-OCH_3_ and H-Si-(DMS)_*m*_-PEG_8_-OCH_3_], with tether lengths (*m*) ~13 to 30 [50]. A silane or triethoxysilane moiety affords crosslinking with addition and condensation cured silicone, respectively. When used to modify silica-filled and unfilled dimethyl silicones, amphiphilic PEG-based SMAs showed appreciable water-driven restructuring for enhanced hydrophilicity and antifouling to various biological foulants [42–44]. Such behavior was also demonstrated for a phenylated silicone system [41]. In contrast, non-amphiphilic PEG-silanes (i.e., no siloxane tether) did not produce this effect. This indicates the important role of the tether to enable surface restructuring, hypothesized to stem from enhanced silicone miscibility and mobility.

While amphiphilic PEG-based SMAs were effective in creating anti-fouling silicones, identification of a PEG-alternative is of interest as recent reports indicate the potential immunogenicity of PEG [51–53]. Poly(2-oxazolines) (POx) have emerged as promising PEG substitutes [54,55]. POx polymers are produced via living cationic ring-opening polymerization (CROP) [56,57]. The POx side-chain and resulting polymer properties are readily tuned based on the 2-substituent of the 2-oxazoline monomer (e.g., alkyl, fluoroalkyl, etc.) [58,59]. Among the properties dictated by the side-chain are water solubility and lower critical solubility temperature (LCST) [60–64]. For poly(2-alkyl-2-oxazoline)s, the hydrophilic poly(2-methyl-2-oxazoline) (PMeOx) and poly(2-ethyl-2-oxazoline) (PEtOx) are particularly promising PEG substitutes [65–67]. The anti-fouling behavior of various POx polymers has been assessed [66,68,69], particularly for grafted surfaces [70]. For instance, PMeOx_*n*_ and PEtOx_*n*_ (e.g., *n* = 100) brushes immobilized on gold and silica surfaces were hydrophilic and reduced protein adsorption [62,71–73]. Reports on bulk modification of silicones with POx-based additives are limited, and have utilized dimethyl silicones but not phenylated silicones [74–76]. Methacrylate graft copolymers comprised of MeOx_n_ or EtOx_n_ (*n* = 10 – 24) and dimethylsiloxane (*m* = 11) were combined at 30 wt% (wt%) with a condensation cure silicone and formed atop a layer of neat silicone [75]. These surfaces demonstrated an increased hydrophilicity and resistance to marine organisms versus unmodified silicone and those prepared with analogous PEG-based copolymers [76]. PMeOx_n_ (*n* ~25) formed with a trimethoxysilane end-group via a C_12_-alkyl sulfide amide ester spacer [(EtO)_3_Si-alkyl-PMeOx], was subsequently combined at 10 wt% with a silica-filled condensation cure silicone [74]. Despite the use of tetrahydrofuran solvent, the limited compatibility of additive in the silicone resulted in increased surface roughness. Additionally, the modified silicone remained hydrophobic and showed no improved resistance to bacteria fouling versus the unmodified silicone.

**Fig. 1.**
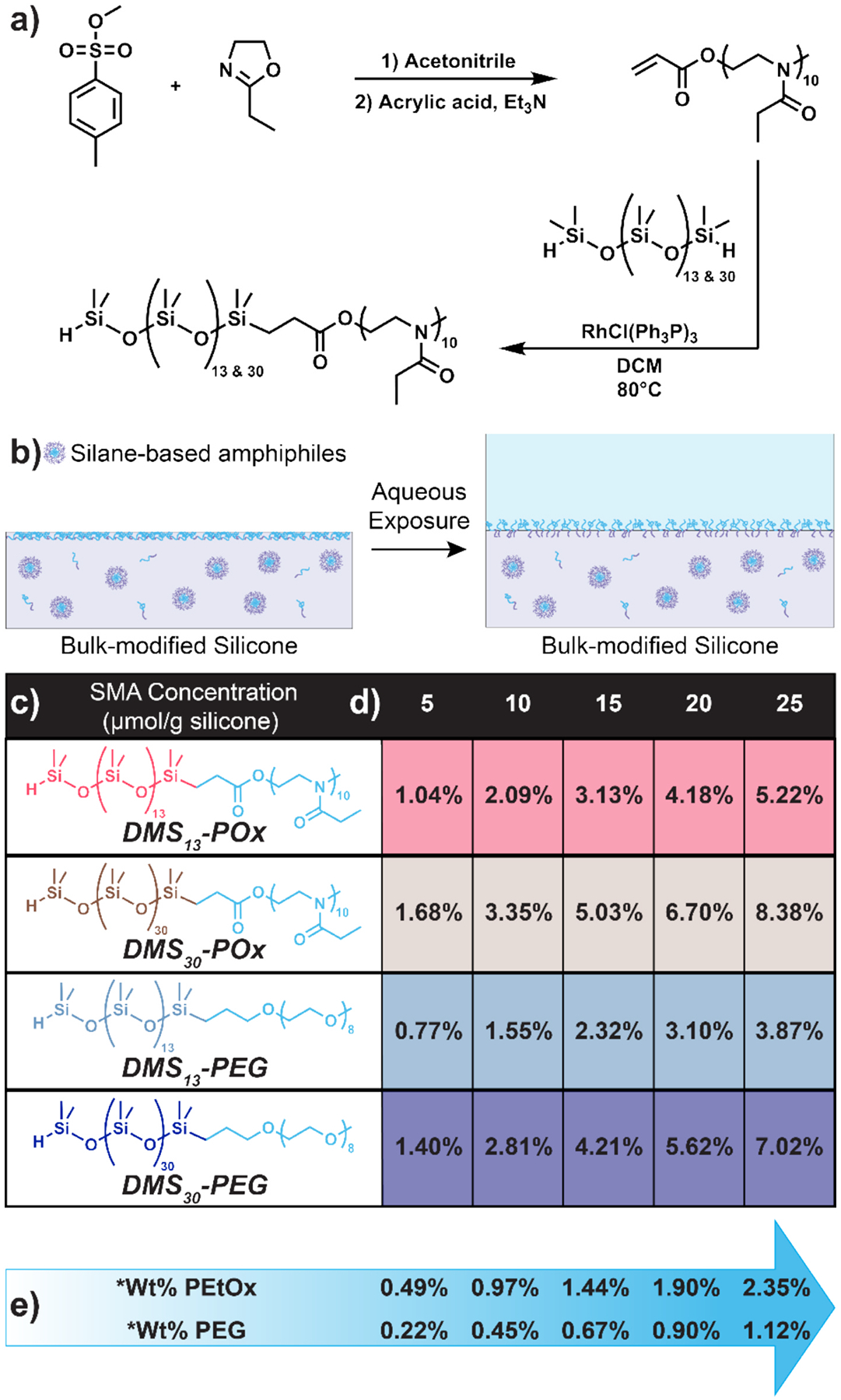
**(a)** Synthesis of POx-silane amphiphiles having a PEtOx segment. **(b)** Depiction of POx- and PEG-silane amphiphiles within a cured silicone before and after restructuring to the surface with aqueous exposure. **(c)** Structures and designations of POx- and PEG-silane amphiphiles as SMAs comprised of siloxane tethers of varying lengths (*m* = 13 and 30). **(d)** Molar concentration of SMAs (5 – 25 μmol g^−1^) introduced into MED-6019 [0% Ph], MED-6010 [5.8% Ph], and MED-6020 [8.5% Ph]. **(e)** For a particular SMA molar concentration, corresponding weight percent of PEtOx or PEG (*wt% w.r.t. silicone).

Herein, amphiphilic PEtOx-based SMAs having siloxane tether were synthesized and combined with unfilled, addition cure silicones of varying wt% phenyl content (Fig. 1). The two POx-silane amphiphiles [“DMS_13_-POx” and “DMS_30_-POx”] were comprised of a crosslinkable silane end group, an oligodimethylsiloxane tether, and a PEtOx segment: H-Si-DMS_*m*_-*block*-PEtOx_10_ (*m* = 13 and 30). Analogous PEG-silane amphiphiles [“DMS_13_-PEG” and “DMS_30_-PEG”]. were also prepared: H-Si-DMS_*m*_-*block*-PEG_8_ (*m* = 13 and 30). Each SMA was combined at 5, 10, 15, 20, and 25 μmol g^−1^ into unfilled (i.e., silica-free) silicones: dimethyl silicone [MED-6019; 0% Ph], dimethyl diphenyl silicone [MED-6010; 5.8% Ph], and dimethyl diphenyl silicone [MED-6020; 8.5% Ph]. To distinguish the effect of siloxane tether length, for a given molar concentration of SMA, the total wt% of PEtOx or PEG is constant. Modified silicones were evaluated in terms of crosslinking, mechanical properties, and water-induced surface restructuring. Human fibrinogen protein adsorption, as well as the accumulation of the fungus *Candida albicans* and several types of bacteria including *Escherichia coli, Pseudomonas aeruginosa, Staphylococcus aureus*, and *Staphylococcus epidermidis* were tested on selected formulations.

## 2. Materials and methods

### 2.1. Materials

2-Ethyl-2-oxazoline, methyl p-toluenesulfonate, anhydrous acetonitrile, triethylamine, acrylic acid, sodium bicarbonate (NaHCO_3_), sodium chloride (NaCl), anhydrous magnesium sulfate (MgSO_4_), tris(triphenylphosphine)rhodium(i) chloride (Wilkinson’s catalyst), hexamethyldisilazane (HMDS), triflic acid, activated charcoal Norit^®^, NMR-grade deuterated chloroform (CDCl_3_) and solvents were purchased from Sigma-Aldrich. Tetramethyldisiloxane (TMDS), octamethylcyclotetrasiloxane (D_4_), and α,ω-bis(SiH)oligodimethylsiloxane_13_ [H-Si-DMS_13_-Si-H, *M*_n_ = 1000 – 1100 g mol^−1^] were obtained from Gelest. Allyl methyl PEG (“allyl-PEG_8_-OCH_3_”) (*M*_n_ = 450 g mol^−1^) was obtained from the NOF Corporation. Allyl-PEG_8_-OCH_3_, reagent-grade dichloromethane (DCM), and CCl_3_ were dried over 4 Å molecular sieves prior to use. Three medical-grade, addition cure silicone elastomers [1:1 Mix Ratio (Part A: Part B)] were purchased from NuSil, having varying wt% phenyl (Ph) content and the following manufacturer’s specifications: **(i)** dimethyl silicone MED-6019 [0 wt% Ph content; Part A: octamethylcyclotetrasiloxane, Part B: 10 – 30 wt% of silicic acid (H_4_SiO_4_), tetraethyl ester, and reaction products with chlorodimethylsilane], **(ii)** dimethyl diphenyl silicone MED-6010 [5.8 wt% Ph content; Part A: octamethylcyclotetrasiloxane, Part B: < 15 wt% of H_4_SiO_4_, tetraethyl ester, and reaction products with chlorodimethylsilane], and **(iii)** dimethyl diphenyl silicone MED-6020 [8.5 wt% Ph content; Part A: octamethylcyclotetrasiloxane, Part B: < 10 wt% H_4_SiO_4_, tetraethyl ester, and reaction products with chlorodimethylsilane]. Phosphate buffered saline (PBS) without calcium and magnesium (pH 7.4) was acquired from Corning. Glass microscope slides (75 × 51 × 1.2 mm) and Fibrinogen Polyclonal Antibody, HRP, Rockland^™^ were purchased from Fisher Scientific. Aluminum panels (4 × 8 × 0.025“) were procured from Q-LAB. Human fibrinogen and Pierce^™^ 1-Step^™^ Ultra TMB-ELISA Substrate Solution were obtained from Avantor. Silica-coated QCM-D sensors (QSX-303) were obtained from Q-Sense. Bacterial and fungal strains were purchased from American Type Culture Collection (ATCC) (Manassas, VA): *Staphylococcus epidermidis* (ATCC 35984), *Staphylococcus aureus* (ATCC 25923), *Escherichia coli* (ATTC 12435), *Pseudomonas aeruginosa* (ATCC 15442), and *Candida albicans* (ATCC 10231). Tryptic Soy broth (TSB), Luria–Bertani broth (LB), minimal medium M63, yeast nitrogen broth (YNB), RPMI 1640 medium, crystal violet powder (CV), 33% glacial acetic acid, 10X PBS, dibasic sodium phosphate (Na_2_HPO_4_), potassium dihydrogen phosphate (KH_2_PO_4_), potassium chloride (KCl), ammonium sulfate ((NH_4_)_2_SO_4_), MgSO_4_, dextrose, thiamine, and biotin were purchased from VWR. Silicone isolator wells for protein adsorption studies were prepared from silicone sheets (*t* ~ 2 mm; McMaster Carr) with a die punch (*d* ~ 18 mm).

### 2.2. Syntheses

All reactions took place under nitrogen (N_2_) with a Teflon-coated stir bar. Chemical structures of products were confirmed via ^1^H NMR spectroscopy using a Bruker Avance Neo 400 MHz spectrometer with an Ascend magnet, an automated tuning 5 mm broadband iProbe, operating in the Fourier transform (FT) mode, and with CDCl_3_ (*δ* = 7.26 ppm) as the standard. ^1^H NMR spectra were utilized to determine number average molecular weight (*M*_n_) values noted below. The *T*_*g*_ of PEtOx_10_-OAc was determined via differential scanning calorimetry (DSC, TA Instruments Q250). Each specimen (~15 mg; *N* = 3) was hermetically sealed before being heated/cooled (10 °C min^−1^) from 0 °C to 100 °C over two cycles. Using the 2nd cycle data, *T*_*g*_ was determined utilizing TA TRIOS software.

#### Synthesis of poly(2-ethyl-2-oxazoline)_10_-acrylate (PEtOx_10_-OAc).

PEtOx_10_-OAc was synthesized via CROP and subsequent acrylation based on prior reports [71,77,78]. 2-Ethyl-2-oxazoline (10.0 mL, 100 mmol) was mixed with acetonitrile (10.0 mL) in a pressured vessel. The solution was sparged with N_2_ for 15 min at room temperature (RT). In a scintillation vial, methyl p-toluenesulfonate (2.328 g, 12.5 mmol) was dissolved in acetonitrile (5.0 mL) and the mixture was added to the flask utilizing a needle and syringe trough a rubber septum. The solution was then sparged with N_2_ for an additional 10 min and purged for 5 min. The reaction was allowed to stir at 80 °C for 2 h. After the CROP was performed, the mixture was allowed to cool to RT and the end-capping agents triethylamine (4.4 mL, 3.13 mmol) and acrylic acid (2.1 mL, 3.13 mmol) were added sequentially to the flask utilizing a needle and syringe through a rubber septum under positive N_2_ pressure. Subsequently, the reaction was stirred at 55 °C overnight (O/N). Acetonitrile was removed via rotary evaporation, and the crude was dissolved in dry CCl_3_. The product was washed with a saturated solution of NaHCO_3_ (15 mL) three times, followed by three washes with a brine solution (15 mL). After O/N separation, the organic layer was collected, dried with MgSO_4_, gravity filtered, solvent removed via rotary evaporation, and dried under vacuum (RT, O/N, 30 in. Hg) to obtain the purified PEtOx_10_-OAc as a yellow solid (7.00 g, 70% yield). ^1^H NMR agreed with that previously reported (*M*_n_ ~991 g mol^−1^) (Fig. S1); *T*_*g*_ = ~48 °C (Table S1, Fig. S2).

#### Synthesis of α,ω-bis-(Si-H)oligodimethylsiloxane_30_ (H-Si-DMS_30_-Si-H).

H-Si-DMS_30_-Si-H was synthesized using triflic acid catalyzed ring-opening polymerizations of D_4_ (33.00 g, 111 mmol) in the presence of TMDS (1.99 g, 15 mmol) as the end-capping agent per a prior report [50]. Reagents were combined in a round-bottom flask (RBF), and 40 μL of triflic acid were added. The mixture was stirred (RT; O/N) and neutralized with the addition of 94 μL of hexamethyldisilazane (RT, 1 h). The crude product was purified by filtration through qualitative filter paper and dried under vacuum to obtain H-Si-DMS_30_-Si-H as a clear, colorless liquid (30.0 g, 86% yield). ^1^H NMR agreed with that previously reported (*M*_n_ ~2359 g mol^−1^) (Fig. S3).

#### Synthesis of H-Si-DMS_13_-block-PEtOx_10_ (DMS_13_-POx).

DMS_13_-POx was synthesized via Wilkinson’s catalyzed (< 10 mg) regioselective hydrosilylation reaction of H-Si-DMS_13_-Si-H (7.36 g, 7 mmol) and PEtOx_10_-OAc (6.64 g, 7 mmol) [1:1 molar ratio]. The reagents were mixed with dry DCM (55 mL) and purged with N_2_ for 5 min stirring at RT in a pressure vessel. After the allotted time, the reaction was allowed to proceed O/N at 80 °C. The catalyst was removed from the reaction by stirring the solution at RT with activated charcoal for 4 h. The crude product was purified by sequential filtration through qualitative and quantitative filter paper, and lastly dried under vacuum (RT, O/N, 30 in. Hg) to obtain purified DMS_13_-POx as a yellow liquid (10 g, 71% yield) (Fig. S4).

#### Synthesis of H-Si-DMS_30_-block-PEtOx_10_ (DMS_30_-POx).

Likewise, DMS_30_-POx was prepared by reacting H-Si-DMS_30_-Si-H (9.86 g, 4 mmol), PEtOx_10_-OAc (4.14 g, 4 mmol), and Wilkison’s catalyst, resulting in purified DMS_30_-POx as a yellow liquid (10 g, 71% yield) (Fig. S5).

#### Synthesis of H-Si-DMS_13_-block-PEG_8_ (DMS_13_-PEG) and H-Si-DMS_30_-block-PEG_8_ (DMS_30_-PEG).

DMS_13_-PEG and DMS_30_-PEG were synthesized as previously reported using a one-step hydrosilylation protocol [50]. Briefly, H-Si-DMS_*m*_-Si-H (*m* = 13 or 30) was reacted with ally-PEG_8_-OCH_3_ (1:1 molar ratio) via a Wilkinson’s catalyzed regioselective hydrosilylation and purified as described above. Both were isolated as clear, colorless liquids. ^1^H NMR spectra agreed with that previously reported (Fig. S6 – S7).

### 2.3. Preparation of silicones modified with DMS_13_-POx, DMS_30_-POx, DMS_13_-PEG, and DMS_30_-PEG

Three addition cure, unfilled silicones of varying phenyl wt% were utilized: dimethyl silicone [MED-6019; 0 wt% Ph], dimethyl diphenyl silicone [MED-6010; 5.8 wt% Ph], and dimethyl diphenyl silicone [MED-6020; 8.5 wt% Ph]. For each silicone type, equal portions of part A and part B were combined. Subsequently, the designated amount of SMA (DMS_13_-POx, DMS_30_-POx, DMS_13_-PEG, or DMS_30_-PEG) was added and mixing achieved with a FlackTek Inc. SpeedMixer^™^ (3500 rpm; 2 min). Resulting silicone blends were prepared as cured specimens as described below.

#### 2.3.1. Preparation of cured silicones

The blended mixture was poured into a one-sided mold consisting of glass slide backing (previously cleaned with isopropyl alcohol) and PTFE mold (50 × 25 × 5 mm; McMaster-Carr). To degas a liquid film, the mold was then placed into a vacuum oven (RT; 1 min; 30 in. Hg) that was vented to 15 in. Hg. This was repeated for five cycles with maximum pressure (30 in. Hg) consecutively held for 2.5, 3.5, 10, 10, and 10 min. To cure a degassed liquid film, the molds were placed in a pre-heated oven at the designated temperature and duration (per manufacturer’s specifications): MED-6019 (50 °C, 2 h); MED-6010 (150 °C, 30 min); and MED-6020 (150 °C, 30 min.). Unmodified MED-6019, MED-6010, and MED-6020 films were likewise prepared as controls. Two grams of a mixture placed into the mold produced films *t* ~1.3 mm × *w* ~25 mm × *l* ~47 mm.

#### 2.3.2. Preparation of disc and tensile specimens

The aforementioned films were carefully peeled from the glass substrate. Discs (*d* ~6 mm × *t* ~1.3 mm) were harvested with a biopsy punch. Disc specimens were utilized for all characterization except for mechanical testing. For tensile testing specimens, films were cut into dog bones (ASTM D1708–18 certified punch). Prior to further testing, free standing discs and tensile specimens were maintained at RT for two weeks.

#### 2.3.3. Preparation of specimens for protein and biofilm formation

Mixtures (4.0 g), analogous to those prepared to fabricate films, were poured onto aluminum panels (previously cleaned with acetone). The uncured mix was spread out with a thin film applicator (GARDCO^®^ Microm II Imperial Film Applicator with anodized aluminum side plates, and standard 400 series stainless-steel blade) to achieve a thickness of 250 μm. Specimens were likewise cured. Discs (*d* ~15 mm × *t* ~250 μm) were harvested with an industrial scale handpress punch.

### 2.4. Silicone characterization

#### 2.4.1. Sol content

The mass of a disc (*N* = 5) was weighed, and each submerged in 10 mL of hexane within a 20 mL scintillation vial. Sealed vials were placed atop a shaker plate (48 h, 150 rpm). Specimens were removed, dried *in vacuo* (60 °C, O/N, 30 in. Hg) and weighed. The initial and final weights of specimens were used to determine % sol content.

#### 2.4.2. Attenuated total reflectance-Fourier transform infrared (ATR-FTIR) spectroscopy

An ALPHA interferometer (Bruker) equipped with a Platinum ATR module (a diamond crystal with a 1.66 μm depth of penetration at 45°) was used to evaluate disc surface composition (*N* = 5) after the sol content test. Each spectrum was obtained in the region of 4000–500 cm^−1^ via 32 scans at a resolution of 4 cm^−1^.

#### 2.4.3. Aqueous-induced mass loss

Disc specimens (*N* = 5) were weighed and submerged in PBS (10 mL), each in a 20 mL scintillation vial, at 37 °C and 120 rpm (VWR Benchtop Shaking Incubator Model 1570). After 2 weeks, specimens were removed, dried *in vacuo* (60 °C, O/N, 30 in. Hg) and weighed. The initial and final weights of specimens were used to determine % mass loss.

#### 2.4.4. Water uptake

Water uptake was measured using thermal gravimetric analysis (TGA; TA instruments Q50). Disc specimens (*N* = 3) were likewise conditioned in PBS for 2 weeks (per section 2.4.3). Upon removal from PBS, a disc was briefly dried under a stream of air, blotted with a paper towel, and placed in its entirety in a platinum TGA pan. The mass change was monitored as the sample was heated from RT to 150 °C under a N_2_ atmosphere, at a rate of 10 °C min^−1^. The water loss was recorded as the peak in the mass loss derivative curve between RT and ~140 °C, coinciding with a loss of water from the specimen. The wt% water uptake was determined by measuring the mass loss percentage over the bounds of this peak.

#### 2.4.5. Mechanical properties

Tensile tests were run in accordance with ASTM D1708–18 and ASTM D638–14. Film dog bone specimens (ASTM D1708–18 certified punch) were placed into an Instron 5944 Universal Testing System equipped with tensile clamps. Specimens (*N* = 5) were strained at a rate of 13 mm min^−1^. From the resulting stress–strain curves, Young’s modulus (*E*) was determined from the slope up to 10% strain using a MATLAB algorithm.

#### 2.4.6. Static water contact angle (θ_static_)

Water-driven surface restructuring of a disc (*N* = 5) was characterized with *θ*_static_ measurements using an Optical Tensiometer (Theta Flex) equipped with an automatic dispenser, video camera, and drop shape analysis software (OneAttension software). A 5 μL deionized water droplet was placed on the disc and *θ*_static_ was iteratively measured over a 3 min period in 15 s intervals. The reported values are an average and standard deviation of measurements made on different regions of different films per sample group.

#### 2.4.7. Protein adsorption

Human fibrinogen adsorption onto silicone films was measured using a modified immunosorbent assay. The coated wells (*N* = 3) of each composition were exposed to 0.15 mL of human fibrinogen solution prepared in PBS (3.0 mg mL^−1^) and statically incubated for 1 h at 37 °C. The protein solution was removed, and each well was rinsed three times with PBS before the addition of TBS-T20 (0.50 mL), which was incubated for 30 min at 37 °C. Wells were then rinsed three times with TBS-T20. Next, 0.5 mL of goat anti-fibrinogen (HRP)-conjugated polyclonal detection antibody (1:50,000 dilution in TBS-T20) were added to each well and statically incubated for 1 h at 37 °C. Wells were then rinsed three times with TBS-T20. TMB di-HCl substrate solution (0.5 mL) was added and allowed to incubate for 30 min at 37 °C. To stop the reaction, 2 M H_2_SO_4_ was added to each well and plates were shaken on an orbital shaker at RT for 15 min. To quantify the amount of adsorbed human fibrinogen on each surface, 0.15 mL of each resulting solution was transferred to a 96-well plate, absorbance was measured at 450 nm using a spectrophotometer (Tecan Safire2), and the value was compared to a human fibrinogen standard curve (0.01 to 10000 ng mL^−1^). The unmodified silicones slides served as hydrophobic control with well-known high protein adsorption.

#### 2.4.8. Biofilm growth and retention

Biofilm growth and retention of bacteria *S. epidermidis*, *S. aureus*, *E. coli*, and *P. aeruginosa* as well as fungal pathogen *C. albicans* on silicone films (*N* = 3) was characterized using a semi-automated, multi-well plate screening methodology [79–81]. Overnight cultures of *S. epidermidis* and *S. aureus* in Tryptic Soy Broth (TSB) were resuspended in 1 × PBS to 0.6 OD_600_ and used to prepare 10^7^–10^8^ cells per mL suspensions in deionized (DI) water supplemented with 10.22 g L^−1^ Na_2_HPO_4_, 3.81 g L^−1^ KH_2_PO_4_, 1.01 g L^−1^ KCl, 0.793 g L^−1^ (NH_4_)_2_SO_4_, 0.06 g L^−1^ MgSO_4_, 0.5 g L^−1^ dextrose, 1.0 μg L^−1^ thiamine and 0.5 L^−1^ μg^−1^ biotin. For *E. coli* and *P. aeruginosa*, overnight cultures were prepared in LB broth, resuspended in 1 × PBS to 0.4 OD_600_ and used to prepare 10^7^–10^8^ cells per mL suspensions in minimal medium M63 + 2 g L^−1^ dextrose (*E. coli*) and 0.6 g L^−1^ TSB in DI water (*P. aeruginosa*). An O/N culture of *C. albicans* in 1 × YNB + 100 mM dextrose was resuspended in 1 × PBS to 0.6 OD_600_ and used to prepare a 10^7^–10^8^ cells per mL suspension in 1:10 RPMI 1640 medium/DI water. One milliliter of the final bacterial and fungal cell suspensions was added to films placed in 24-well plates [79] and incubated statically at 37 °C for 72 h for *S. aureus* and *S. epidermidis* and 24 h for the remaining microorganisms to promote cell attachment and biofilm growth. The films were rinsed 3 × with 1 × PBS, dried at ambient laboratory conditions for 1 h and stained with 0.5 mL of a CV dye solution (0.35% in DI water) for 15 min. Excess CV solution was removed and films were rinsed 3 × with DI water, dried for 1 h at ambient laboratory conditions and extracted in 0.5 mL of 33% glacial acetic acid for 15 min to solubilize CV dye bound to retained biofilm. A 0.15 mL aliquot of acetic acid extract from each silicone film was transferred to 96-well plate and measured for absorbance at 600 nm using a multi-well plate spectrophotometer. Three replicates for each composition were normalized to an assay control (i.e., growth media without microorganism) and the average value was reported for each microorganism.

### 2.5. Statistical analysis

Reported values represent an average and standard deviation of *N* ≥ 3 measurements. Statistical analysis was performed using two factor ANOVA followed by Tukey’s multiple comparison and statistical significance was set at a *p*-value < 0.05.

## 3. Results and discussion

### 3.1. Synthesis of POx- and PEG-based silane amphiphile SMAs

#### 3.1.1. Synthesis of poly(2-ethyl-2-oxazoline)_10_-acrylate (PEtOx_10_-OAc)

PEtOx_10_-OAc was prepared with modifications of previously reported syntheses via a CROP of 2-ethyl-2-oxazoline with methyl p-toluenesulfonate as the initiator in acetonitrile [71,77,78]. The reaction was terminated by a mixture of acrylic acid and triethylamine to yield the acrylate end functionalized PEtOx. End group analysis (NC*H*_*3*_, *δ* = 2.90–3.07 ppm) was used to confirm *M*_n_ and degree of polymerization [*M*_n_ = 991 g mol^−1^; *n* ~10] (Fig. S1). DSC revealed that PEtOx_10_-OAc displayed a *T*_*g*_ of ~48 °C (Table S1, Fig. S2). Owing to its low molecular weight, this value was expectedly somewhat lower than that reported for PEtOx (e.g., 50 kg mol^−1^, *T*_*g*_ ~70 °C) [63,64]. Additionally, at this low molecular weight (*n* = 10), PEtOx LCST is known to be absent.

#### 3.1.2. Synthesis of H-Si-DMS_30_-Si-H

Triflic acid-catalyzed ring-opening reaction of D_4_ with TMDS was used to prepare H-Si-DMS_30_-Si-H as previously reported [50]. ^1^H NMR confirmed the chemical structure, and *M*_n_ via end group analysis (Fig. S3).

#### 3.1.3. Synthesis of DMS_13_-POx and DMS_30_-Pox

Synthesis of POx- and PEG-based silane amphiphiles utilized regioselective hydrosilylation of disilanes using Wilkinson’s catalyst. This approach is known to form monosubstituted products, owing to the enhanced reactivity of one Si-H terminus of α,ω-bis(Si-H)-terminated compounds toward vinyl-containing compounds [82,83]. POx-silane amphiphiles were prepared with two siloxane tether lengths (*m* = 13 and 30). Regioselective hydrosilylation of PEtOx_10_-OAc with H-Si-DMS_13_-Si-H and H-Si-DMS_30_-Si-H successfully produced DMS_13_-POx and DMS_30_-POx, respectively. DCM was employed as the solvent based on poor solubility of PEtOx_10_-OAc in toluene. ^1^H NMR confirmed the chemical structures (Figs. S4 – S5).

#### 3.1.4. Synthesis of DMS_13_-PEG and DMS_30_-PEG

PEG-silane amphiphiles with two siloxane tether lengths (*m* = 13 and 30) were likewise prepared per our prior reports [50]. In another prior study, we also confirmed that regioselective hydrosilylation yielded the targeted monosubstituted DMS_13_-PEG [83]. Regioselective hydrosilylation of ally-PEG_8_-OCH_3_ with H-Si-DMS_13_-Si-H and H-Si-DMS_30_-Si-H in toluene yielded DMS_13_-PEG and DMS_30_-PEG, respectively. ^1^H NMR confirmed the chemical structures (Fig. S6 – S7).

### 3.2. Preparation of SMA-modified silicones

The ability of PEtOx-silane amphiphiles (DMS_13_-POx and DMS_30_-POx) to serve as SMAs for addition cure silicones of varying phenyl content was evaluated as follows. DMS_13_-POx and DMS_30_-POx were each combined at five concentrations (5, 10, 15, 20, and 25 μmol g^−1^) into three unfilled (i.e., silica-free), addition cure silicones [MED-6019 (0 wt % Ph), MED-6010 (5.8 wt% Ph), and MED-6020 (8.5 wt% Ph)]. Notably, these silicones are ‘unfilled’ or ‘non-reinforced’ as they lack silica filler. No solvent was added to create the silicone mixtures. It was noted that the PEtOx_10_-OAc (i.e., no ‘DMS’ tether) was immiscible and could not be directly mixed into the silicones (i.e., insoluble in the silicone mixtures). In contrast, despite the lack of a solvent, DMS_13_-POx and DMS_30_-POx formed homogeneous mixtures with all silicones at all concentrations, as did DMS_13_-PEG and DMS_30_-PEG. This miscibility is attributed to the siloxane tether of the SMAs. At a given molar concentration of DMS_13_-POx and DMS_30_-POx, the wt% of PEtOx was held constant (Fig. 1). For instance, at 5 μmol g^−1^, there is 0.49 wt% of PEtOx_10_ for both PEtOx-based SMAs. Likewise, for DMS_13_-PEG and DMS_30_-PEG, the wt% of PEG was held constant at a molar concentration. This allowed the effect of the DMS tether length (*m* = 13 or 30) to be directly probed.

### 3.3. Crosslinking and aqueous stability

#### 3.3.1. Crosslinking – sol content and ATR-FTIR

As addition cure silicones, MED-6019, −6010, and −6020 undergo crosslinking via hydrosilylation reactions between vinyl and silane (Si-H) moieties. The silane moiety of DMS_13_-POx and DMS_30_-POx, as well as DMS_13_-PEG and DMS_30_-PEG, should afford crosslinking within the silicone network. Sol content studies were used to confirm that the incorporation of the SMAs did not inhibit a silicone’s ability to cure (Table S2). After soaking films in hexane for 48 h, all modified silicones maintained sol contents of < ~10%, indicative of sufficient crosslinking, with subtle increases generally observed with higher SMA concentrations. ATR-FTIR spectra were also acquired for the sol-extracted films as well as the neat POx- and PEG-based SMAs (Fig. S8 – S27). At all SMA concentrations (5 – 25 μmol g^−1^), the modified silicones displayed a characteristic silane peak (2280–2080 cm^−1^) like that of the corresponding unmodified silicone. The silane peak did not increase with greater SMA concentration, indicating sufficient excess vinyl moieties in these 2-part silicones that can accommodate (i.e., crosslink with) the additional silane from the SMA.

#### 3.3.2. Aqueous-induced mass loss and water uptake

Given their anticipated dwelling in physiological environments, the impact of conditioning silicones modified with amphiphilic POx- and PEG-based SMAs in PBS at 37 °C for two weeks was assessed. All modified silicone specimens exhibited mass losses < 1% and most were statistically similar to the corresponding unmodified silicone, indicative of stability to the aqueous environment (Table S3). As determined by TGA, water uptake by modified silicones was very low (< 1%) (Fig. S28, Table S4). Thus, despite the introduction of hydrophilic PEtOx and PEG segments from the SMAs, the silicones do not become appreciably hydroscopic, even as the SMA content is increased.

### 3.4. Mechanical properties

**Fig. 2.**
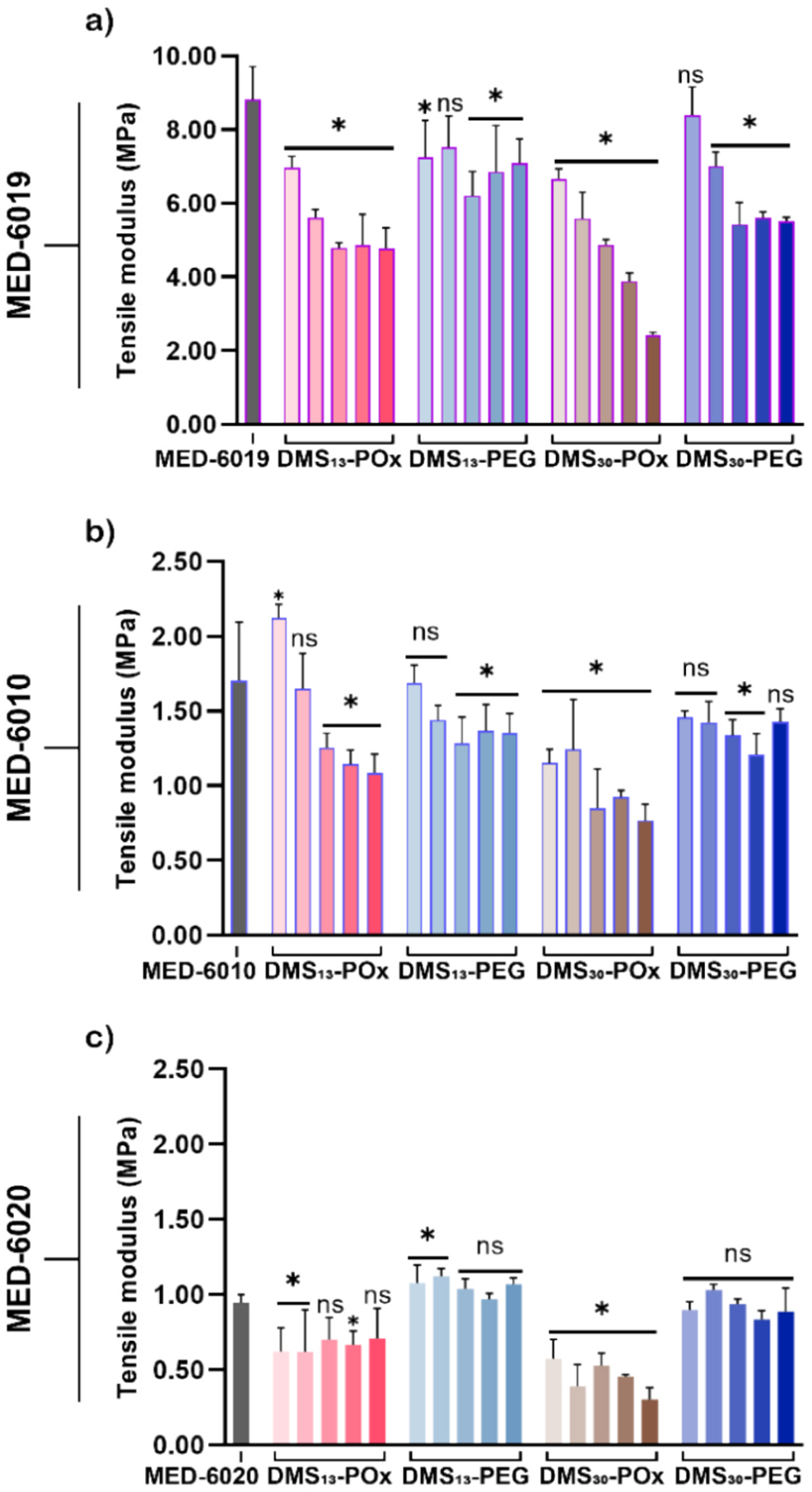
Tensile modulus of **(a)** MED-6019 [0% Ph], **(b)** MED-6010 [5.8% Ph], and **(c)** MED-6020 [8.5% Ph] unmodified and SMA-modified films. For a given SMA, bars indicate increasing concentration from left to right (5 – 25 μmol g^−1^). * p < 0.05 vs corresponding unmodified silicone. DMS_13_-PEG and DMS_30_-PEG on MED-6020 data reported by Marmo et al. [41].

As noted above, the crosslinking of silane-functionalized amphiphilic POx- and PEG-based SMAs in addition cure silicones was achieved. However, the POx or PEG termini remain as dangling free ends (Fig. S29), and may impart plasticization effects leading to softening of the modified silicone [84]. Indeed, plasticization of silicones is frequently observed with the addition of low molecular weight silicones or organic molecules such as PEG [85,86]. Prior reports have yielded mixed results on plasticizing effects. No appreciable plasticization was observed for a silica-filled, condensation cure dimethyl silicone (MED-1137) modified with analogous triethoxysilylpropyl-terminated amphiphilic PEG-based SMAs [42]. However, some plasticization was observed for the unfilled, dimethyl diphenyl silicone (MED-6020; 8.5% Ph) modified with DMS_13_-PEG and DMS_30_-PEG [41]. Thus, the tensile properties of all SMA-modified silicones were evaluated and compared to the respective unmodified silicones (Fig. 2, Table S5). For these unfilled, unmodified silicones, the tensile modulus decreased with phenyl content: MED-6019 (0% Ph) [~8.8 MPa] > MED-6010 (5.8% Ph) [~1.7 MPa] > MED-6020 (8.5% Ph) [~0.9 MPa] (Fig. 2a). In the case of dimethyl silicone (MED-6019), incorporation of DMS_13_-POx and DMS_30_-POx at all concentrations (≥ 5 μmol g^−1^) led to significant decreases in moduli, and was notably progressive for DMS_30_-POx of increasing concentration. For DMS_13_-PEG in particular, as well as for DMS_30_-PEG, relatively reduced decreases in moduli were produced. A somewhat similar trend was observed for dimethyl diphenyl MED-6010 (Fig. 2b). Moduli were decreased at somewhat only higher concentrations of DMS_13_-POx (≥ 15 μmol g^−1^) but at all concentrations of DMS_30_-POx (≥ 5 μmol g^−1^). DMS_13_-PEG produced reductions in moduli at concentrations ≥ 15 μmol g^−1^, similar to DMS_13_-POx. In the case of DMS_30_-PEG, a lack of or only subtle decreases in moduli were observed, even at the highest concentration (25 μmol g^−1^). For dimethyl diphenyl MED-6020, DMS_13_-POx produced moduli similar to the unmodified silicone at 15 and 25 μmol g^−1^ but reduced moduli at the other concentrations (Fig. 2c). DMS_30_-POx resulted in decreased moduli at all concentrations. In the case of DMS_13_-PEG, moduli increased somewhat at low concentrations (5 and 10 μmol g^−1^), but were similar for higher concentrations (≥ 15 μmol g^−1^). DMS_30_-PEG produced no change in moduli across all concentrations. Analogous trends were observed for reductions in tensile strength (Fig. S30, Table S6). As expected, decreases in tensile modulus and strength generally coincided with increased strain at break (Fig. S31, Table S7). Overall, the extent of SMA-induced plasticization was dependent on the phenyl content of the silicone. The silicone with the highest phenyl content [MED-6020 (8.5% Ph)] was relatively the least impacted by the addition of SMAs. In contrast, the dimethyl silicone [MED-6019 (0% Ph)] exhibited greater plasticization, even at lower SMA concentrations. The SMA type also had a distinct plasticizing effect, and was generally greater for POx-based SMAs versus PEG-based SMAs. As noted above, PEG-based SMAs did not produce plasticization in a silica-filled RTV silicone [42]. These collective results suggest that silica filler may mitigate plasticization effects imparted by POx-based as well as PEG-based SMAs, and could be incorporated into these unfilled silicones for this purpose if desired.

### 3.5. Contact angle analysis

**Fig. 3.**
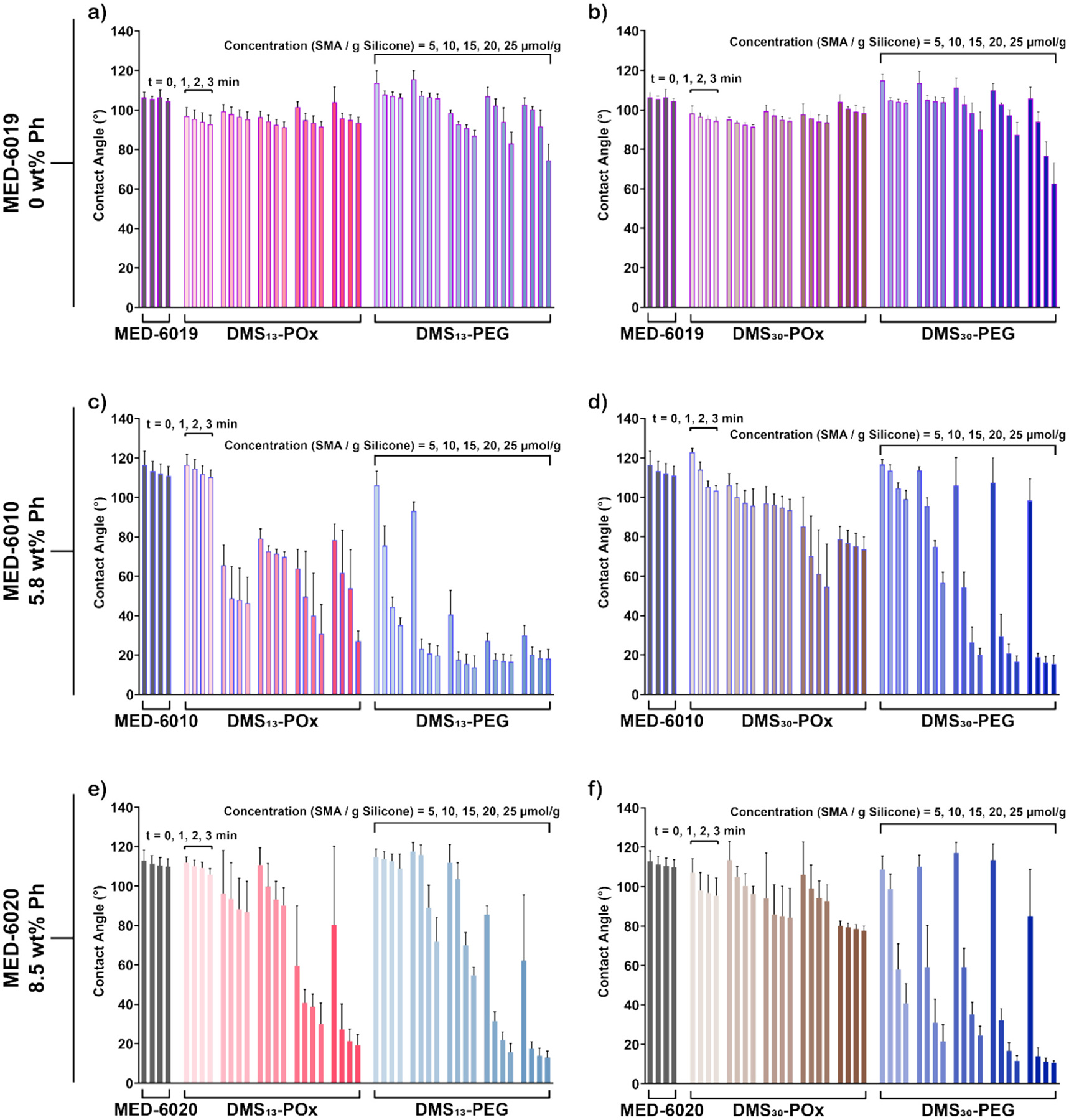
Contact angle (θ_static_) at 0, 1, 2, and 3 min for unmodified and modified silicones: **(a, b)** MED-6019, **(c, d)** MED-6010, and **(e, f)** MED-6020 each with DMS_13_-POx, DMS_13_-PEG, DMS_30_-POx, and DMS_30_-PEG (5 – 25 μmol g^−1^). MED-6020 modified with DMS_13_-PEG and DMS_30_-PEG data previously reported by Marmo et al. [41].

The efficacy of PEtOx-silane amphiphiles (DMS_13_-POx and DMS_30_-POx) as SMAs to produce anti-fouling silicones critically rests on their ability to rapidly and substantially restructure to the aqueous interface and form a PEtOx-rich surface. Water-driven restructuring of SMAs can be assessed by temporal contact angle analysis of water droplets deposited onto the surfaces of modified silicone. Surface restructuring would be marked by a high initial contact angle (*θ*_static,0 min_ > 100°) characteristic of silicones, followed by a substantial decrease over 3 min (*θ*_static,3 min_ ≪ 100°). This behavior has been previously noted in the case of PEG-silane amphiphiles (DMS_13_-PEG and DMS_30_-PEG) incorporated into various dimethyl silicones but notably absent in the case of PEG-silane (i.e., no siloxane tether) [42,44–46]. For DMS_13_-PEG and DMS_30_-PEG, the water-driven restructuring behavior was attributed to the siloxane tether and resulting amphiphilic, diblock nature. It was also previously noted that DMS_*m*_-PEG SMAs formed micelles (~200 – 300 nm) in a hydrophobic solvent, and that PEG could be detected at the air interface of cured silicones [87]. Thus, such PEG-based SMAs are thought to reside at the interface as unimers, as well as in micelles, with a dynamic exchange known to occur for diblock oligomers in general [88–90]. Absent a siloxane tether, PEG is unable to distribute in this fashion within the silicone, leading to macroscopic phase separation that precludes mobilization to the aqueous interface. For amphiphilic PEtOx-based SMAs incorporated into silicones, the presence of a siloxane tether was likewise expected to lead to water-driven surface restructuring. However, the distinct nature of PEtOx versus PEG is notable and may lead to a distinct effect. PEtOx is amphiphilic due to the ethyl group, and this may impact restructuring behavior of DMS_13_-POx and DMS_30_-POx. Although PEtOx is known to have an LCST, it is well above RT (e.g., ~69 °C as for PEtOx_500_) and is furthermore absent for lower molecular weights (e.g., PEtOx_10_) like that of the amphiphilic PEtOx-based SMAs. Still, within a micelle core, the hydrophobic associations of the ethyl group of the PEtOx segment may reduce the exchange with surface unimers and water-driven restructuring versus that of PEG. The ethyl groups may further reduce the hydrophilicity of a PEtOx-enriched surface versus a PEG-enriched surface. To probe this behavior, modified silicone surfaces with varying concentrations of SMAs (5 – 25 μmol g^−1^) were subjected to contact angle analysis of deposited water droplets over a period of 3 min (Fig. 3).

In the case of the dimethyl silicone MED-6019 (0 wt% Ph), limited restructuring was observed when modified with PEtOx-based SMAs (Fig. 3a–b, Table S8). Even at the highest concentration (25 μmol g^−1^) of DMS_13_-POx and DMS_30_-POx, modified silicones remained hydrophobic (*θ*_static,3min_ ~94° and ~98°, respectively) with negligible reduction from the corresponding *θ*_static,0min_ (~105°). Silicones modified with PEG-based SMAs displayed some restructuring at concentrations ≥ 10 μmol g^−1^, achieving *θ*_static,3min_ value of ~75° (DMS_13_-PEG) and ~63° (DMS_30_-PEG) at 25 μmol g^−1^. Greater restructuring was previously observed when PEG-based SMAs were incorporated into a silica filled, condensation cure dimethyl silicone (MED-1137) at 25 μmol g^−1^, for both silane-terminated [*θ*_static,2min_ ~50° (DMS_13_-PEG) and ~43° (DMS_30_-PEG)] and also triethoxysilane-terminated [*θ*_static,2min_ ~59° (DMS_13_-PEG) and ~58° (DMS_30_-PEG)] [45]. This suggests that the silica filler may favorably disrupted SMA solubility within the dimethyl silicone. Thus, it is hypothesized that in dimethyl silicone MED-6019, the POx- and PEG-based SMAs are highly soluble (i.e., miscible) due to their chemically similar dimethyl siloxane tethers, reducing the thermodynamic drive of SMA PEtOx and PEG segments to the aqueous interface. As noted above, the amphiphilic nature of the PEtOx segment may contribute to relatively reduced surface hydrophilicity for POx-based SMAs.

Distinct surface behavior was seen for dimethyl diphenyl silicones modified with amphiphilic POx- and PEG-based SMAs (Fig. 3c–d, Table S9). For MED-6010 (5.8 wt% Ph), DMS_13_-POx at concentrations ≥ 10 μmol g^−1^ produced initial contact angle values (*θ*_static,0min_ ~79 – 66°) notably lower versus the unmodified silicone (*θ*_static,0min_ ~117°). This suggests that the SMA had organized towards the air interface to some extent during curing. Indicative of substantial water-driven restructuring, the contact angle decreased over time, particularly at higher concentrations (20 and 25 μmol g^−1^) to achieve values of *θ*_static,3min_ ~28°. DMS_30_-POx at similar concentrations (≥ 10 μmol g^−1^) produced relatively higher initial contact angles (*θ*_static,0min_ ~106 – 79°), indicating less presence at the air interface. There was also less of a decrease in contact angle over time, including at 20 and 25 μmol g^−1^ (*θ*_static,3min_ ~65°). Thus, a shorter siloxane tether (*m* = 13) improved the restructuring of POx-based SMAs to the aqueous interface over the course of 3 min. In comparison, PEG-based SMAs showed greater water-driven restructuring overall. For DMS_13_-PEG, even the lowest concentrations exhibited a large reduction in contact angle (5 μmol g^−1^: *θ*_static,0min_ ~106° to *θ*_static,3min_ ~35°; 10 μmol g^−1^: *θ*_static,0min_ ~93° to *θ*_static,3min_ ~20°). At higher concentrations (15 – 25 μmol g^−1^) of DMS_13_-PEG, the initial contact angles were low (*θ*_static,0min_ ~41 – 27°), suggesting SMA organization to the air interface, before ultimately achieving *θ*_static,3min_ values of ~20°. For DMS_30_-PEG at concentrations ≥ 10 μmol g^−1^, surfaces exhibited the typical transition from high initial contact angles (*θ*_static,0min_ ~114 – 98°) to very low contact angles, particularly at 20 and 25 μmol g^−1^ (*θ*_static,0min_ ~16°). Somewhat similar trends were observed for MED-6020 (8.5% Ph) (Fig. 3e–f, Table S10). For DMS_13_-POx, at 5 – 15 μmol g^−1^, minimal water driven restructuring was observed [*θ*_static,0min_ ~106° and *θ*_static,3min_ ~94°]. Higher concentrations (20 and 25 μmol g^−1^) led to lower contact angles (*θ*_static,3min_ ~25°). For DMS_30_-POx, modest water-driven restructuring was observed across all concentrations. For DMS_13_-PEG at 10 and 15 μmol g^−1^, significant water driven restructuring was observed [*θ*_static,0min_ ~115° and *θ*_static,3min_ ~63°]. At higher concentrations (20 and 25 μmol g^−1^), greater hydrophilicity was achieved although initial contact angles were also reduced somewhat [*θ*_static,0min_ ~74° and *θ*_static,3min_ ~15°]. Finally, all concentrations of DMS_30_-PEG produced substantial water-driven restructuring, and *θ*_static,3min_ values of ~40 – 10° were observed.

These contact angle analyses point to the substantial impact of phenyl content of unfilled silicones in the ability of amphiphilic POx- and PEG-based SMAs to induce water-driven restructuring to form hydrophilic surfaces. Overall, both types of SMAs were least effective in the dimethyl silicone (MED-6019; 0% Ph). Prior work indicates that the inclusion of silica filler could enhance the restructuring behavior of the SMAs. For dimethyl diphenyl silicones [MED-6010 (5.8% Ph) and MED-6020 (8.5% Ph)], DMS_13_-PEG and DMS_30_-PEG were very effective at most concentrations ≥ 10 μmol g^−1^. The length of the siloxane tether had a notable effect, with DMS_30_-PEG more effective in terms of achieving hydrophilicity from an initially hydrophobic surface (i.e., limited organization at the air interface). In contrast, a shorter siloxane tether was advantageous to POx-based SMAs, and DMS_13_-POx (≥ 10 μmol g^−1^) was able to produce highly hydrophilic surfaces while DMS_30_-POx did not. DMS_13_-POx also appeared to restructure more to the air interface in MED-6010 versus MED-6020.

### 3.6. Modified silicones – anti-biofouling behavior

**Fig. 4.**
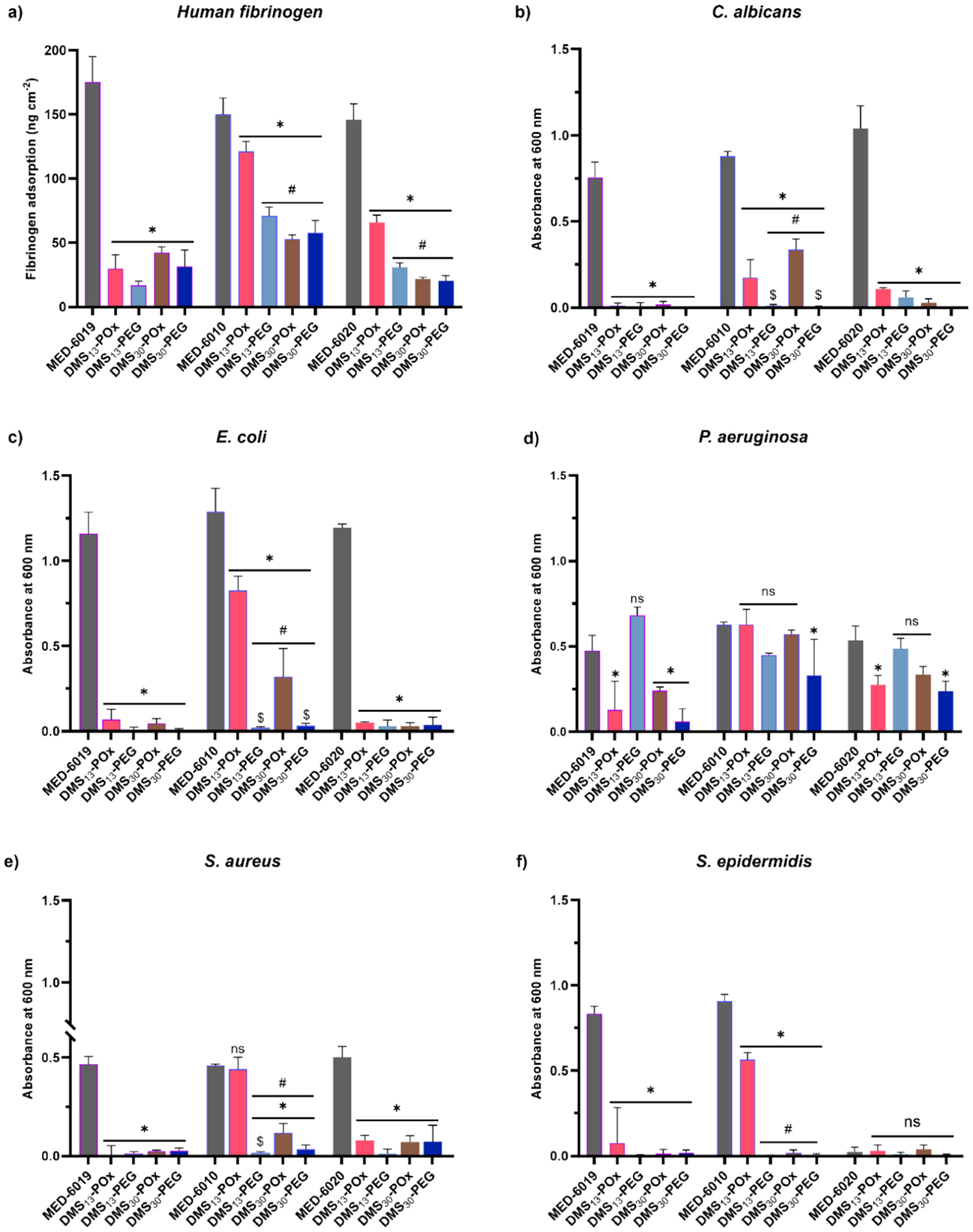
**(a)** Human fibrinogen adsorption, **(b)** fungal biofilm formation, and **(c-f)** bacterial biofilm formation on unmodified silicones and silicones bulk-modified with POx- and PEG-based SMAs. * *p* < 0.05 vs corresponding unmodified silicone. # *p* < 0.05 vs DMS_13_-POx. $ *p* < 0.05 vs DMS_30_-POx.

The broad-spectrum anti-biofouling behavior of silicones modified with amphiphilic PEtOx-silane versus PEG-silane was evaluated in terms of resistance to protein, fungus, and bacteria as these play key roles in thrombosis and infection. For these analyses, each silicone [MED-6019 (0% Ph); MED-6010 (5.8% Ph); MED-6020 (8.5% Ph)] was modified with 25 μmol g^−1^ of a given SMA. In the case of human fibrinogen, adsorption onto modified silicones was evaluated by exposure to a human fibrinogen solution at 37 °C for 1 h (Fig. 4a, Table S11). Owing to their hydrophobicity, all unmodified silicones absorbed substantial quantities of human fibrinogen. Each SMA produced significant reductions in human fibrinogen absorption versus the corresponding unmodified silicone. For dimethyl silicone [MED-6019; 0% Ph], all SMAs led to similarly reduced human fibrinogen levels. However, in the dimethyl diphenyl silicones [MED-6010 (5.8% Ph) and MED-6020 (8.5% Ph)], DMS_13_-POx produced less resistance to human fibrinogen versus DMS_30_-POx as well as PEG-based SMAs. Fungal and bacteria biofilm growth and retention was evaluated via incubation with a designated suspension at 37 °C for 24 or 72 h followed by brief rinsing. For *C. albicans* fungus, the biofilm was significantly reduced for all silicones modified with each SMA (Fig. 4b). In the dimethyl silicone [MED-6019; 0% Ph] as well as dimethyl diphenyl silicone [MED-6020; 8.5% Ph], all SMAs reduced fungal adsorption to negligible levels. However, for dimethyl diphenyl silicone [MED-6010; 5.8% Ph], DMS_13_-POx and DMS_30_-POx led to greater biofilms versus DMS_13_-PEG and DMS_30_-PEG, respectively. For the gram-negative bacteria *E. coli*, all SMAs reduced biofilm formation for all silicones (Fig. 4c). Similar to that observed for *C. albicans*, in dimethyl diphenyl silicone [MED-6010; 5.8% Ph], DMS_13_-POx and DMS_30_-POx led to greater biofilms versus DMS_13_-PEG and DMS_30_-PEG, respectively. For the gram-negative *P. aeruginosa*, several formulations notably did not produce a reduction in biofilm versus unmodified silicones (Fig. 4d). DMS_13_-POx and DMS_30_-PEG were both effective in the dimethyl silicone [MED-6019; 0% Ph] and dimethyl diphenyl silicone [MED-6020; 8.5% Ph]. Only DMS_30_-PEG was effective in dimethyl diphenyl silicone [MED-6010; 5.8% Ph]. For the gram-positive *S. aureus* and *S. epidermis*, SMAs produced a reduction in biofilm formation versus unmodified silicones (Fig. 4e,f). The exception was DMS_13_-POx in the dimethyl silicone [MED-6019; 0% Ph], which led to similar *S. aureus* versus the unmodified silicone. Levels of *S. epidermis* on this particular formulation were also higher versus MED-6019 modified with all other SMAs.

Overall, both PEtOx- and PEG-based SMAs improved the anti-fouling behavior of modified silicones. The extent of fouling reduction was dependent on phenyl content of the silicone, with SMA-modified dimethyl diphenyl silicone [MED-6010; 5.8% Ph] generally exhibiting the highest relative levels of fouling. In MED-6010, DMS_13_-POx showed reduced efficacy against most foulants versus the other SMAs. In all silicones, DMS_30_-POx mostly showed similar effectiveness versus PEG-based SMAs. Interestingly, the anti-fouling results of silicones modified with PEtOx-based SMAs were not entirely predicted by contact angle analyses (i.e., *θ*_static,0 min_ > 100° and *θ*_static,3 min_ ≪ 100°) (Fig. 3). For instance, while DMS_13_-POx and DMS_30_-POx in dimethyl silicone [MED-6019 (0% Ph)] did not produce substantial water-driven restructuring versus PEG-based SMAs, high anti-fouling behavior was achieved. Likewise, in dimethyl diphenyl silicones [MED-6010 (5.8% Ph) and MED-6020 (8.5% Ph)], DMS_13_-POx showed greater restructuring versus DMS_30_-POx, yet the latter produced better anti-fouling. This suggests that water-driven restructuring may continue beyond 3 min, leading to greater POx-enrichment at the aqueous interface. Still, DMS_13_-POx and particularly DMS_30_-POx appear to be highly effective in reducing fouling to dimethyl and dimethyl diphenyl silicones.

## 4. Conclusion

Both dimethyl and dimethyl diphenyl silicones are utilized to create various medical devices, but suffer from poor anti-fouling behavior due to their hydrophobic surfaces. In prior work, we established that siloxane tethered PEG amphiphiles could act as effective SMAs in silicones, enabling water-driven restructuring to the aqueous interface for repulsion of foulants. PEtOx represents a potential anti-fouling alternative to PEG, which has recently been the subject of growing concerns (e.g., immunogenicity). Herein, PEtOx-based silane amphiphiles with two siloxane tether lengths (DMS_13_-POx and DMS_30_-POx) were synthesized as SMAs. Each was incorporated (5 – 25 μmol g^−1^) into three unfilled, addition cure silicones of varying phenyl wt% [MED-6019 (0% Ph), MED-6010 (5.8% Ph), and MED-6020 (8.5% Ph)]. For comparison, silicones were likewise modified with analogous PEG-based silane amphiphile SMAs (DMS_13_-PEG and DMS_30_-PEG). Owing to the silane (Si-H) end group of the SMA, silicone crosslinking was not compromised. Additionally, all modified silicones showed stability in water, as noted by a lack of water uptake and mass loss. In the case of mechanical and surface properties, including anti-fouling behavior, the silicone type (i.e., phenyl content) had a distinct impact. Following crosslinking, the PEtOx and PEG segments are present as dangling ends within the silicone networks, and may induce plasticization. Dimethyl silicone [MED-6019 (0% Ph)] exhibited the greatest reductions in tensile modulus by SMAs overall, while highest phenyl content [MED-6020 (8.5% Ph)] was relatively the least impacted. Plasticization was also generally more pronounced for POx-based SMAs versus PEG-based SMAs. The inclusion of silica, a well-known strategy to reinforce silicones, is considered a plausible tactic that could be employed to compensate for SMA-induced plasticization. Contact angle analyses point to the substantial impact of phenyl content of unfilled silicones in the ability of amphiphilic POx- and PEG-based SMAs to induce water-driven restructuring to form hydrophilic surfaces. Overall, both types of SMAs were least effective in the dimethyl silicone MED-6019 (0% Ph). For dimethyl diphenyl silicones [MED-6010 (5.8% Ph) and MED-6020 [8.5%]), DMS_13_-POx produced highly hydrophilic surfaces at sufficient concentrations (20 and 25 μmol g^−1^) whereas those with DMS_30_-POx were less hydrophilic. Both PEG-silane SMAs led to high hydrophilicity (≥ 10 μmol g^−1^). The ability of POx-based versus PEG-based SMAs to create anti-fouling silicones was assessed against a broad spectrum of fouling species commonly encountered in medical devices, including human fibrinogen protein, *C. albicans* fungus, and 4 bacteria. For these analyses, each silicone was modified with a SMA at 25 μmol g^−1^. With the dimethyl silicone [MED-6019; 0% Ph], POx-based SMAs produced a similarly substantial reduction in human fibrinogen adsorption as well as fungal and bacteria biofilm formation versus PEG-based SMAs. The exception was DMS_13_-POx with respect to *P. aeruginosa*. For the dimethyl diphenyl silicones with the highest phenyl content [MED-6020; 8.5% Ph], DMS_30_-POx produced a reduction of human fibrinogen similar to that of PEG-based SMAs, but DMS_13_-POx produced less of a reduction. POx-based SMAs produced a similar reduction versus PEG-based SMAs for *C. albicans*, *E. coli*, *S. aureus*, and *S. epidermis*. For *P. aeruginosa*, only DMS_13_-POx and DMS_30_-PEG led to reductions versus unmodified MED-6020, and were also similar to each other. For the other dimethyl diphenyl silicones [MED-6010; 5.8% Ph], all POx-based SMAs led to a significant reduction in fouling versus unmodified silicone, except for *P. aeruginosa* and *S. aureus*. DMS_30_-POx produced similar reductions in human fibrinogen and *S. epidermis* versus PEG-based SMAs. For *E. coli* and *S. aureus*, DMS_30_-POx was more effective than DMS_13_-POx, but less effective than PEG-based SMAs. For *C. albicans*, DMS_13_-POx was more effective than DMS_30_-POx. In the case of *P. aeruginosa*, only DMS_30-_PEG led to a significant decrease in biofilm versus the unmodified MED-6010. Overall, POx-based SMAs generally reduced fouling versus unmodified silicones. DMS_30_-POx often achieved similar fouling reductions versus PEG-based SMAs. This is interesting as DMS_30_-POx produced less water-driven surface hydrophilicity versus DMS_13_-PEG and also PEG-based SMAs, according to contact angle analyses of water droplets over a period of 3 min. This suggests that water-driven restructuring may continue beyond 3 min, leading to greater POx-enrichment at the aqueous interface. Thus, contact angle analysis alone cannot fully predict the extent of anti-fouling behavior. This study collectively suggests that amphiphilic POx-based SMAs, having a siloxane tether and PEtOx segment, can induce anti-fouling behavior to silicones having various phenyl content. It is noted that introduction of reinforcing silica filler may impact, potentially favorably, some properties of the resulting SMA-modified silicones. Future studies of these amphiphilic coatings will include evaluating the impact of silica filler on key properties (e.g., mechanical properties and water-driven restructuring). Furthermore, substitution of the PEtOx segment with a more hydrophilic segment [e.g., PMeOx or poly(oxazine)] could further improve anti-fouling behavior.

## Supplementary Material

Support Information

## Data Availability

Statement Raw data is available via the Texas Data Repository (TDR).

## Appendix A. Supplementary data

^1^H NMR spectra; DSC data; sol content data; ATR-FTIR spectra; mass loss data; water uptake data; tensile mechanical properties, contact angle data; fibrinogen adsorption data; crystal violet absorbance data; crystal violet absorbance images. Supplementary data to this article can be found online at https://doi.org/10.1016/j.eurpolymj.2026.114638.
