# Supplementary material for: Siloxane-tethered poly(2-ethyl-2-oxazoline) as surface modifying additives for anti-fouling silicones
Source: Eur Polym J. Author manuscript; Available in PMC 2026 May 19. (PMC13183407; doi:10.1016/j.eurpolymj.2026.114638)
Supplement: Support Information [file NIHMS2175605-supplement-Support_Information.pdf]

Supporting Information

Siloxane-tethered poly(2-ethyl-2-oxazoline) as surface modifying additives for anti-fouling silicones

Jenlyan Negrón Hernández[1], Anika S. Palacharla[2], Darian K. Kanu[3], Shane J. Stafslien[4], Lyndsi Vander Wal,[4] Melissa A. Grunlan[1,2,3]

[1]Department of Chemistry, Texas A&M University, College Station, Texas 77843, United States.

[2]Department of Biomedical Engineering, Texas A&M University, College Station, Texas 77843, United States.

[3]Department of Materials Science and Engineering, Texas A&M University, College Station, Texas 77843, United States.

[4]Department of Coatings and Polymeric Materials, North Dakota State University, Fargo, North Dakota 58108, United States.

*Corresponding author email: mgrunlan@tamu.edu

35 Pages

32 Figures

12 Tables

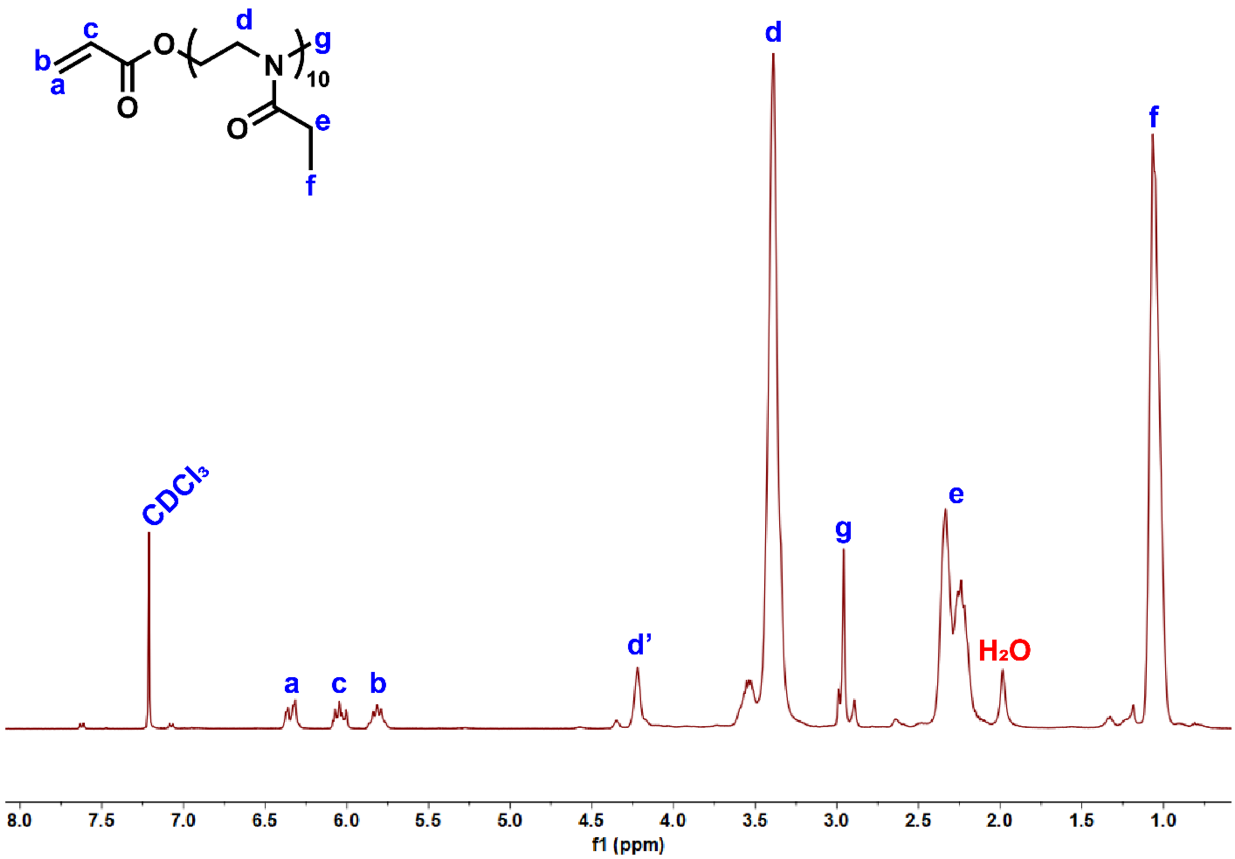

Figure S1. ^{1}H-NMR of PEtOx$_{10}$-OAc (CDCl$_3$; δ, ppm): 0.94-1.20 (m, 30H, CH$_2$C*H$_3$*), 2.12-2.58 (m, 20H, C*H$_2$*CH$_3$), 2.90-3.07 (s, 3H, NC*H$_3$*), 3.16-3.84 (m, 44H, NC*H$_2$*C*H$_2$*), 5.76-5.96 (t, 1H, CH=C*H*=CH), 5.99–6.22 (m, 1H, OCC*H*=CH$_2$), and 6.29–6.51 (m, 1H, C*H*=CH=CH).

Table S1. DSC analysis of $PEtOx_{10}$-OAc.

	T_g (°C)
**$PEtOx_{10}$-OAc**	48.2 ± 0.4

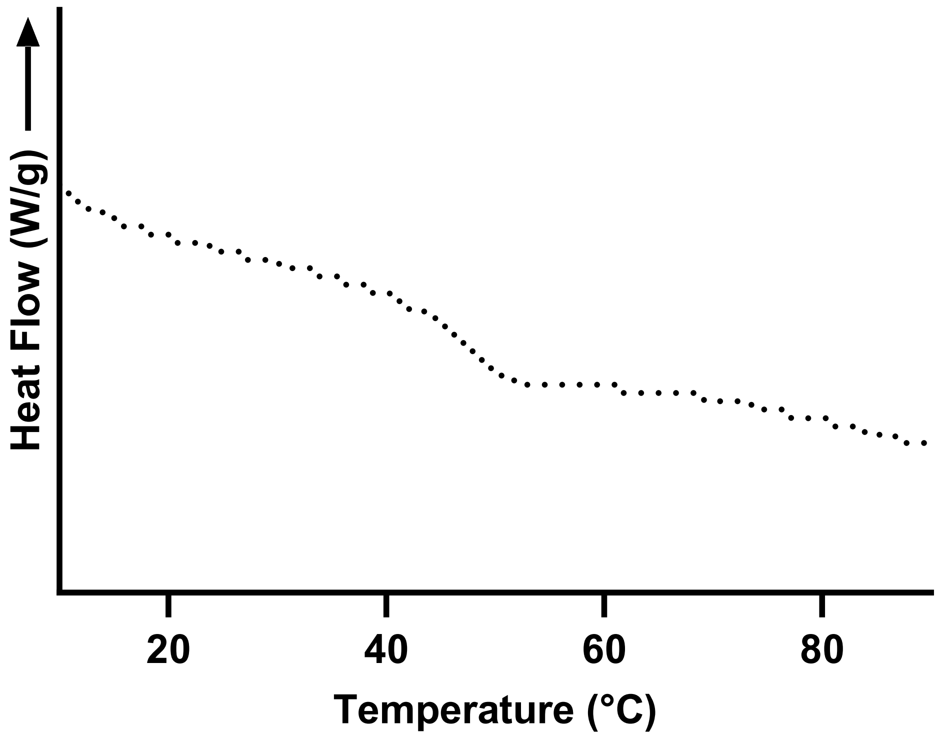

Figure S2. DSC thermogram of $PEtOx_{10}$-OAc.

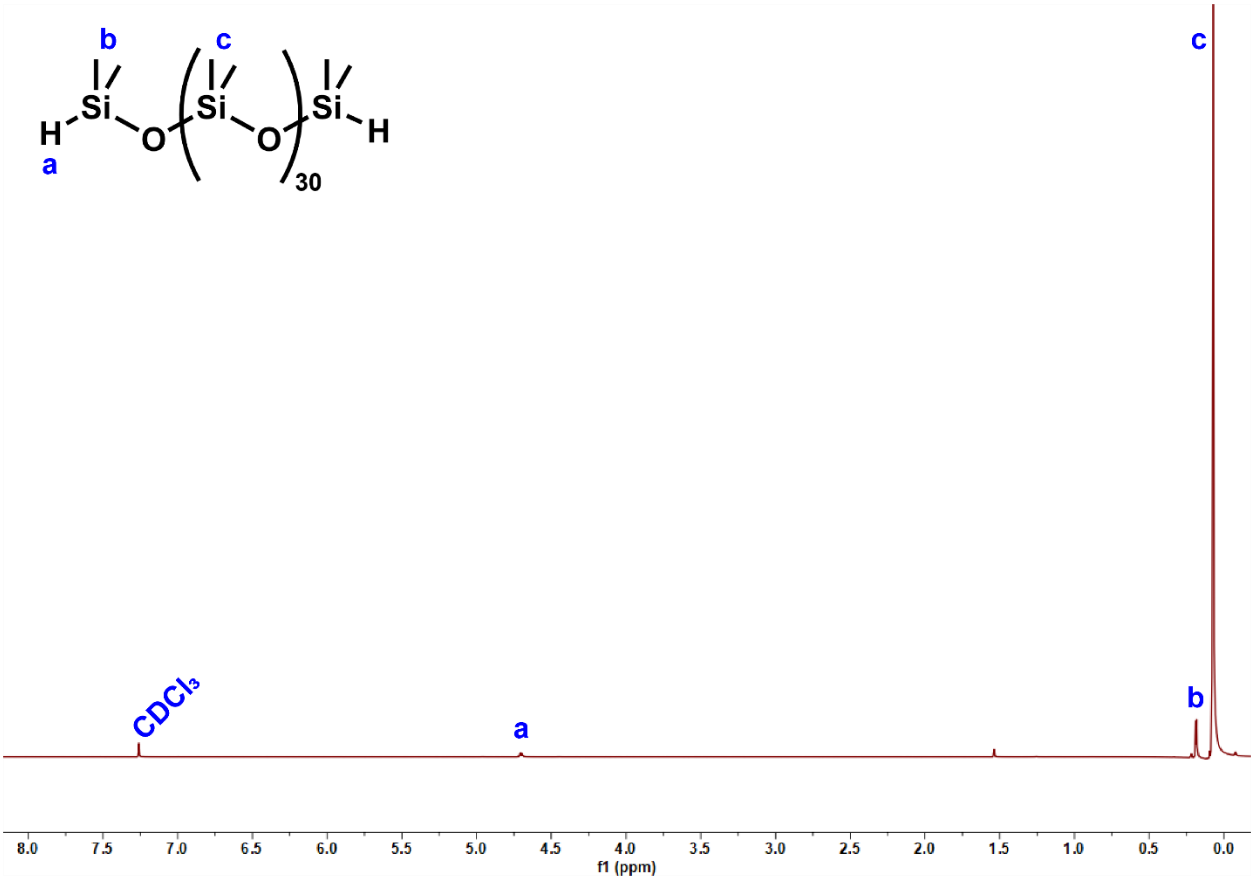

Figure S3. ^{1}H-NMR of H-Si-DMS$_{30}$-Si-H ($CDCl_3$; δ, ppm): 0.02-0.12 (m, 180H, SiCH_3), 0.17-0.20 (d, 12H, OSi[CH_3]$_2$H), and 4.60-4.80 (m, 2H, SiH).

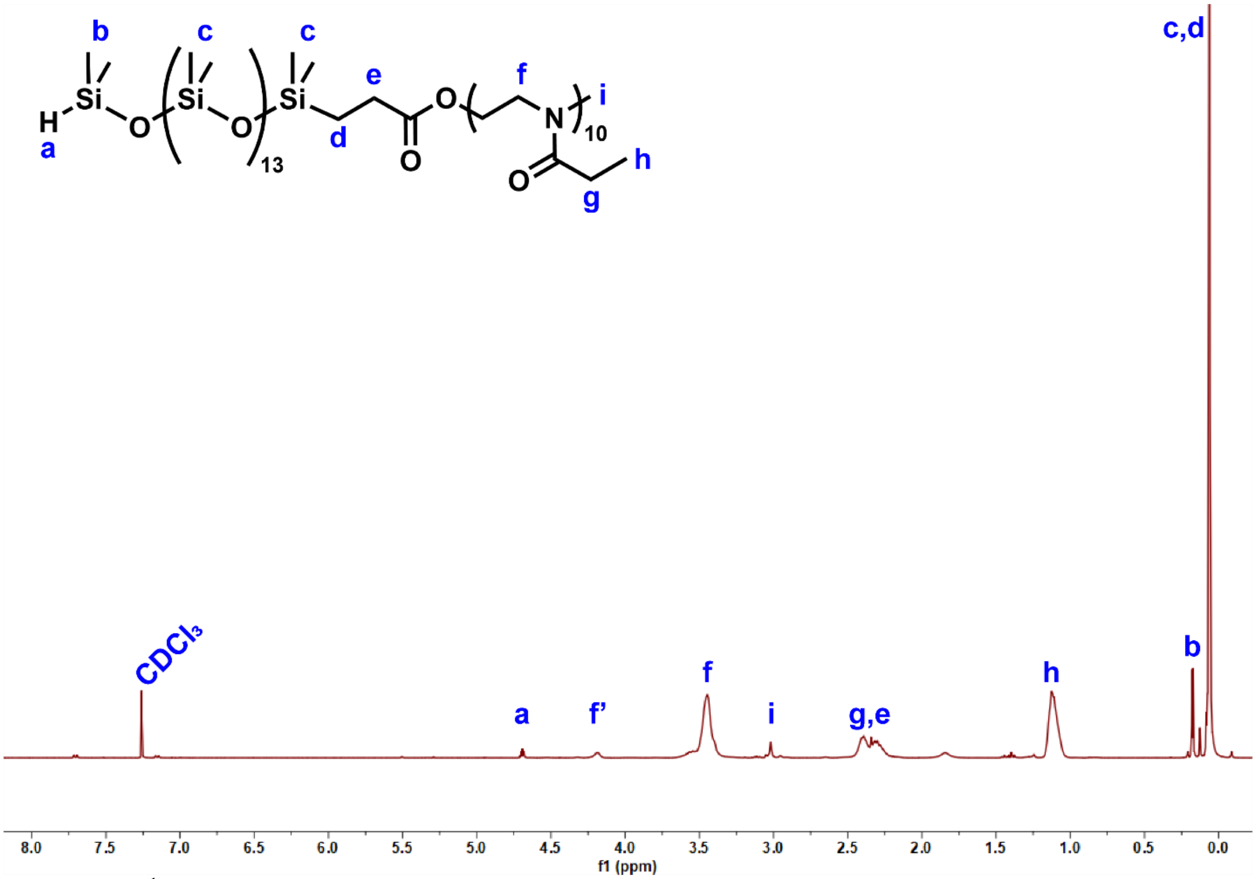

Figure S4. ^{1}H-NMR of DMS_{13}-POx ($CDCl_3$; δ, ppm): 0.01-0.13 (m, 86H, Si*CH_3* and Si*CH_2*CH_2), 0.16-0.19 (d, 6H, OSi[*CH_3*]$_2$H), 1.02-1.19 (t, 30H, NCOCH_2*CH_3*), 2.22-2.50 (m, 22H, NCO*CH_2* and OCO*CH_2*), 2.91-3.07 (s, 1H, *CH_3*-NCO$CH_2$$CH_3$), 3.28-3.69 (m, 44H, N*$CH_2$*$CH_2$*), 4.60-4.80 (m, 1H, SiH).

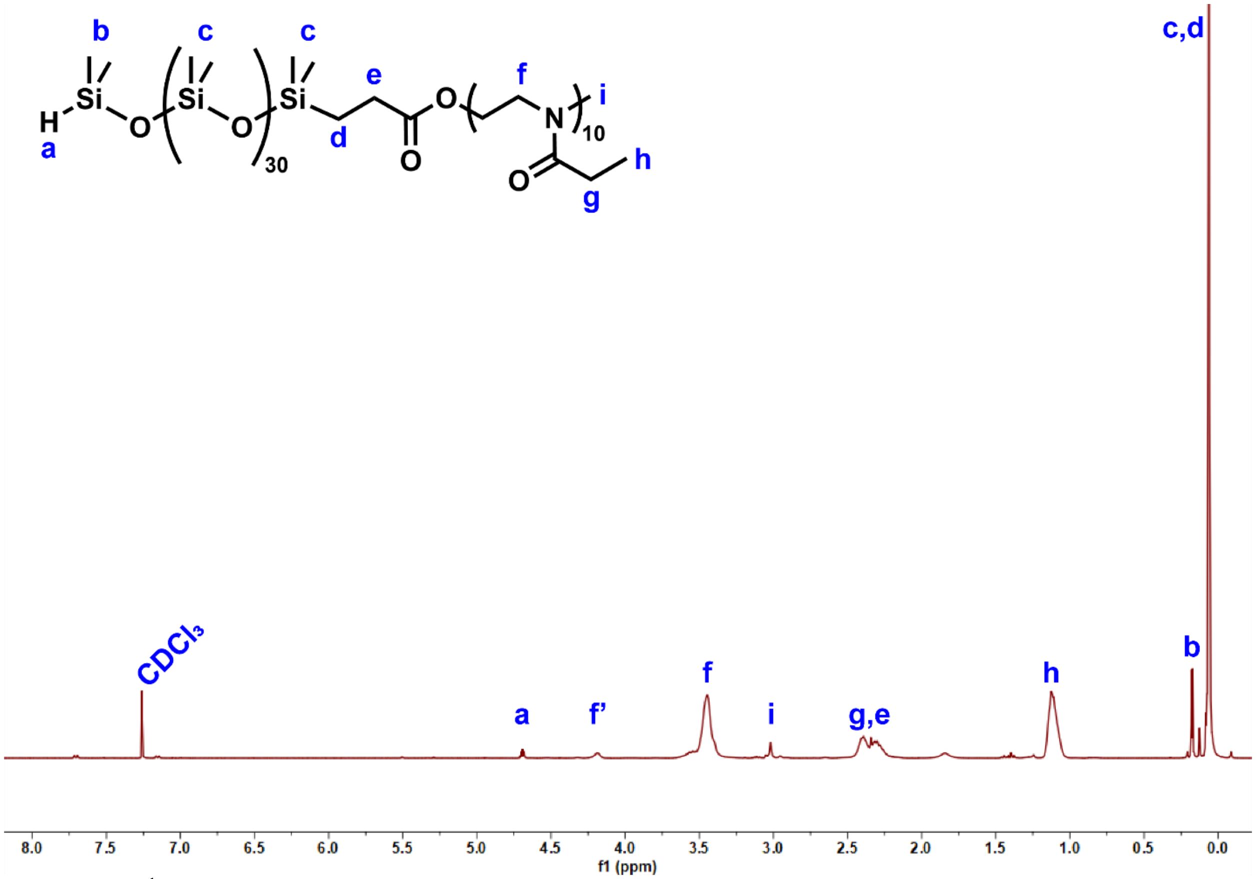

Figure S5. ^{1}H-NMR of DMS$_{30}$-POx ($CDCl_3$; δ, ppm): 0.01-0.13 (m, 188H, SiCH_3 and Si$CH_2$$CH_2$), 0.16-0.19 (d, 6H, OSi[CH_3]$_2$H), 1.02-1.19 (t, 30H, NCO$CH_2$$CH_3$), 2.22-2.50 (m, 22H, NCOCH_2 and OCOCH_2), 2.91-3.07 (s, 1H, CH_3-NCO$CH_2$$CH_3$), 3.28-3.69 (m, 44H, N$CH_2$$CH_2$), 4.60-4.80 (m, 1H, SiH).

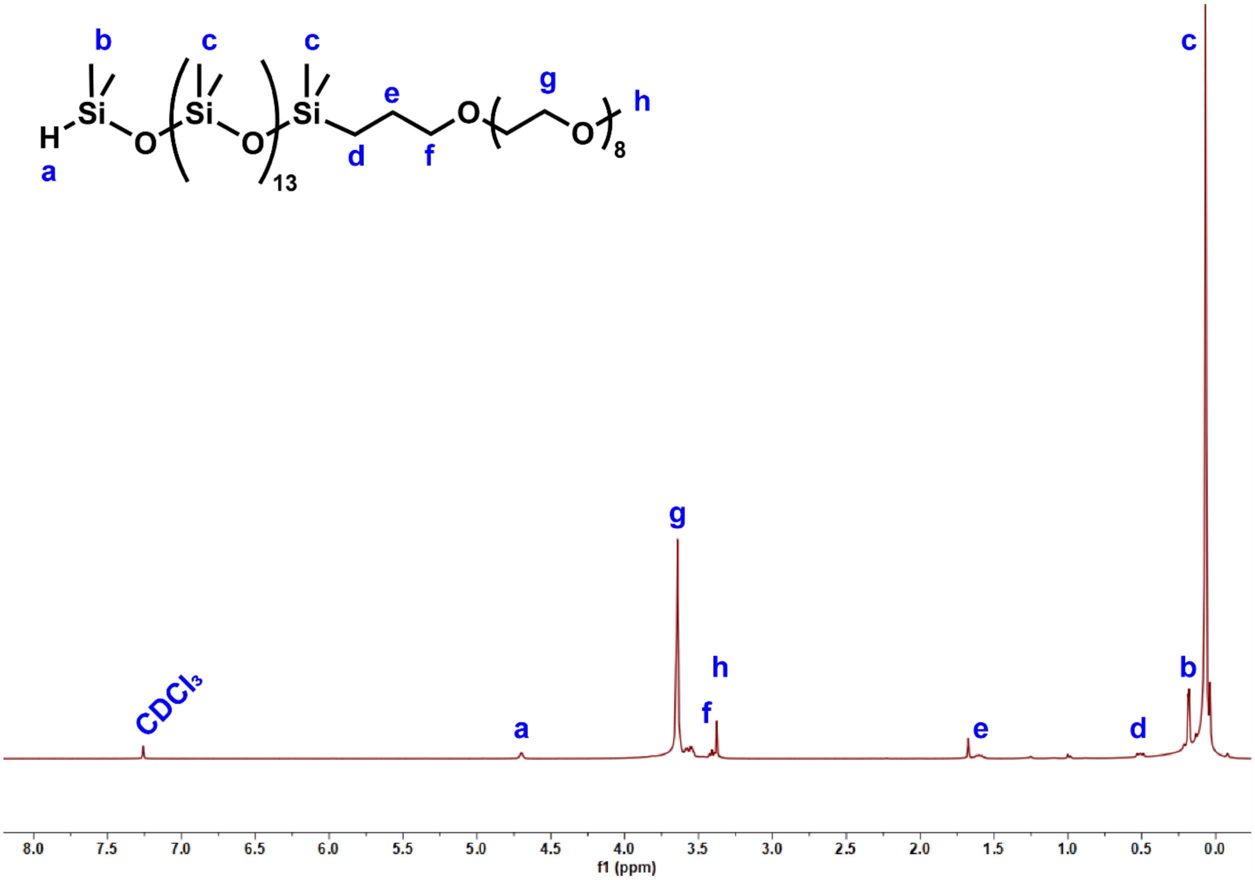

Figure S6. ^{1}H-NMR of DMS$_{13}$-PEG ($CDCl_3$; δ, ppm): 0.01-0.13 (m, 84H, SiCH_3), 0.16-0.19 (d, 6H, OSi[CH_3]$_2$H), 0.46-0.56 (m, 2H, SiCH_2CH$_2$CH$_2$) 1.48-1.63 (m, 2H, SiCH$_2$CH_2CH$_2$), 3.38 (s, 3H, OCH_3), 3.39-3.45 (t, 2H, SiCH$_2$CH$_2$CH_2), 3.50-3.90 (m, 34H, OCH_2CH_2), 4.60-4.80 (m, 1H, SiH).

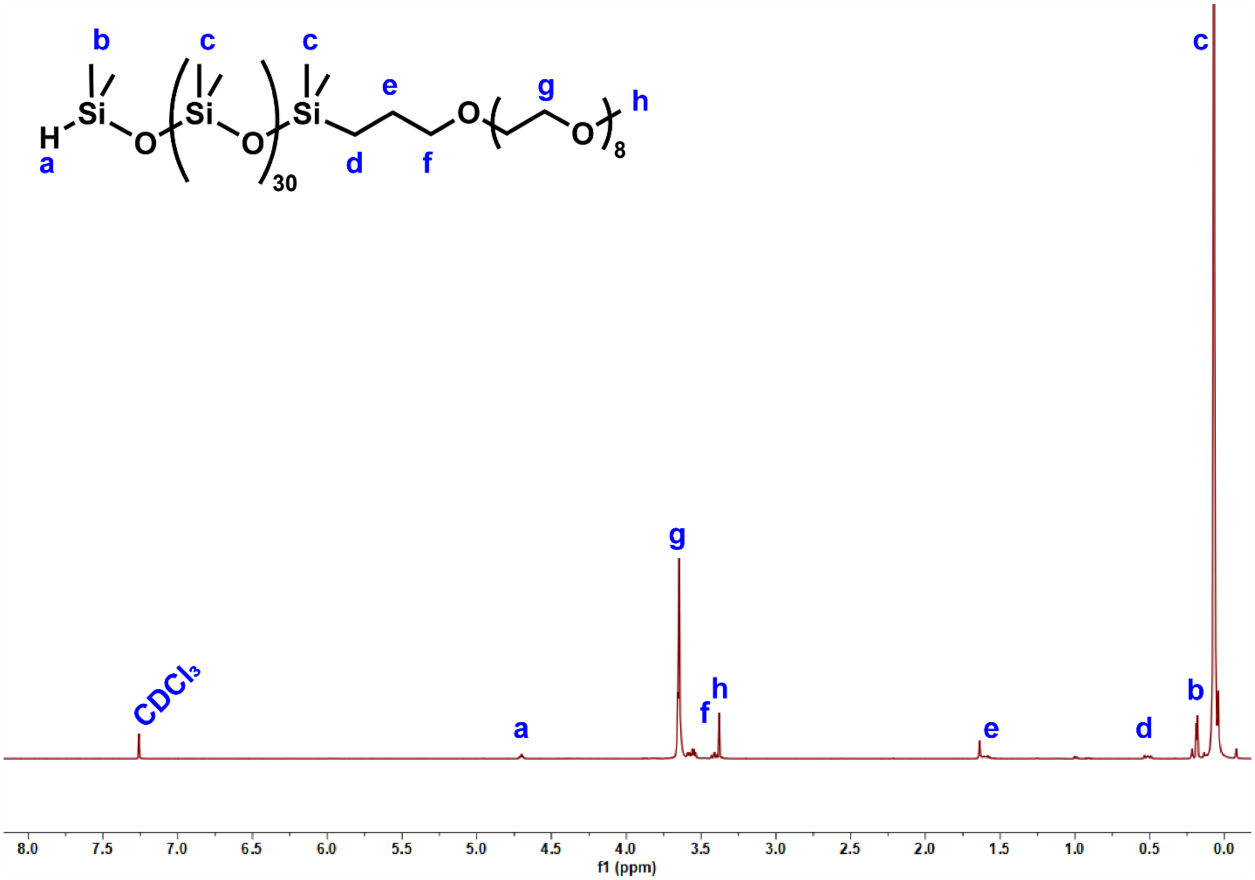

Figure S7. ^{1}H-NMR of DMS_{30}-PEG ($CDCl_3$; δ, ppm): 0.01-0.13 (m, 186H, SiCH_3), 0.16-0.19 (d, 6H, OSi[CH_3]$_2$H), 0.46-0.56 (m, 2H, Si$CH_2CH_2CH_2$) 1.48-1.63 (m, 2H, $SiCH_2CH_2CH_2$), 3.38 (s, 3H, OCH_3), 3.39-3.45 (t, 2H, $SiCH_2CH_2CH_2$), 3.50-3.90 (m, 34H, OCH_2CH_2), 4.60-4.80 (m, 1H, SiH).

Table S2. Sol content of unmodified silicones (MED-6019, -6010, and -6020) and silicones modified with POx and PEG-based SMAs.

	Concentration (µmol g^{-1})	MED-6019 [0% Ph] Sol content (%)	MED-6010 [5.8% Ph] Sol content (%)	MED-6020 [8.5% Ph] Sol content (%)
**Unmodified**	---	4.50 ± 0.56	3.53 ± 0.93	6.31 ± 1.73
**DMS$_{13}$-POx**				
	5	5.04 ± 0.81	5.05 ± 2.21	5.70 ± 0.36
	10	5.07 ± 0.33	7.40 ± 1.16*	7.09 ± 0.49
	15	5.97 ± 0.39*	7.23 ± 1.08*	6.24 ± 0.30
	20	6.44 ± 0.73*	8.15 ± 1.90*	7.06 ± 0.68
	25	7.13 ± 0.34*	10.40 ± 1.81*	8.80 ± 0.75*
**DMS$_{30}$-POx**				
	5	4.93 ± 0.54	6.55 ± 0.29*	6.05 ± 1.01
	10	6.67 ± 0.25*	6.82 ± 0.80*	6.27 ± 0.29
	15	7.32 ± 0.25*	8.04 ± 0.74*	7.69 ± 0.33
	20	9.76 ± 0.50*	8.92 ± 0.78*	8.17 ± 0.64*
	25	10.68 ± 0.28*	8.86 ± 1.02*	8.42 ± 0.97*
**DMS$_{13}$-PEG**				
	5	5.51 ± 0.58*	6.16 ± 0.94*	4.34 ± 1.21 ᵻ
	10	5.67 ± 0.48*	6.98 ± 1.16*	5.15 ± 0.63 ᵻ
	15	8.75 ± 0.10*	7.09 ± 0.57*	4.22 ± 1.19 ᵻ
	20	6.93 ± 0.73*	6.78 ± 0.93*	4.76 ± 0.62 ᵻ
	25	8.38 ± 0.29*	7.86 ± 1.13*	4.31 ± 0.53 ᵻ
**DMS$_{30}$-PEG**				
	5	4.70 ± 0.60	6.14 ± 0.20*	4.09 ± 0.60 ᵻ
	10	5.95 ± 0.29*	5.87 ± 1.39*	4.67 ± 0.53 ᵻ
	15	7.22 ± 0.80*	7.26 ± 0.27*	5.39 ± 0.69 ᵻ
	20	7.76 ± 0.14*	6.99 ± 0.32*	5.02 ± 0.62 ᵻ
	25	8.34 ± 0.42*	8.24 ± 0.13*	5.18 ± 0.13 ᵻ

ᵻ Data reported by Marmo et al. [1]. * $p < 0.05$ vs corresponding unmodified silicone.

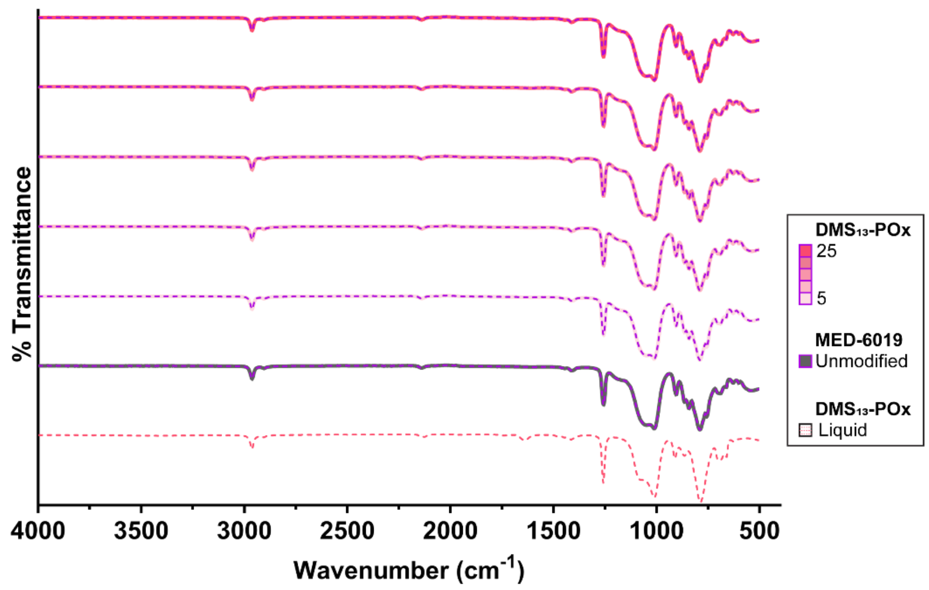

Figure S8. ATR-FTIR spectra of unmodified silicone (MED-6019; 0% Ph) and silicones modified with DMS_{13}-POx at all concentrations (5 – 25 µmol g^{-1}). Color gradient (top of legend) refers to concentration of DMS_{13}-POx (µmol g^{-1}) added to silicone.

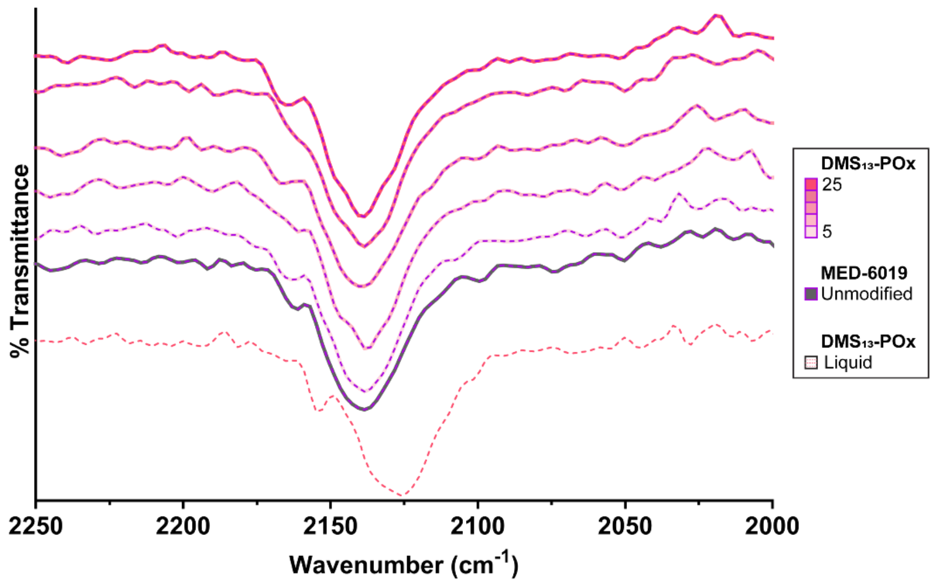

Figure S9. ATR-FTIR spectra of unmodified silicone (MED-6019; 0% Ph) and silicones modified with DMS_{13}-POx at all concentrations (5 – 25 µmol g^{-1}). Color gradient (top of legend) refers to concentration of DMS_{13}-POx (µmol g^{-1}) added to silicone. Characteristic Si-H peak at 2280-2080 cm^{-1}.

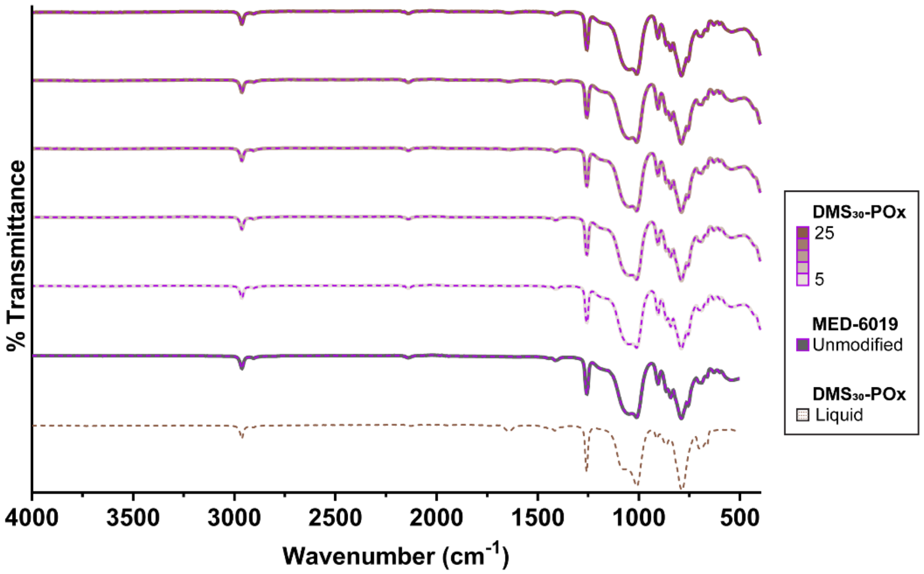

Figure S10. ATR-FTIR spectra of unmodified silicone (MED-6019; 0% Ph) and silicones modified with DMS_{30}-POx at all concentrations (5 – 25 µmol g^{-1}). Color gradient (top of legend) refers to concentration of DMS_{30}-POx (µmol g^{-1}) added to silicone.

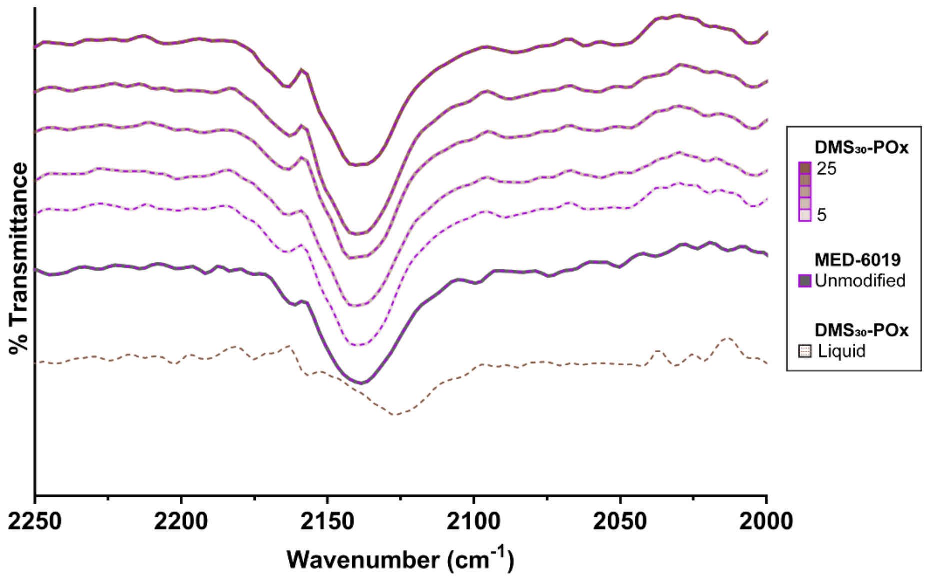

Figure S11. ATR-FTIR spectra of unmodified silicone (MED-6019; 0% Ph) and silicones modified with DMS_{30}-POx at all concentrations (5 – 25 µmol g^{-1}). Color gradient (top of legend) refers to concentration of DMS_{30}-POx (µmol g^{-1}) added to silicone. Characteristic Si-H peak at 2280-2080 cm^{-1}.

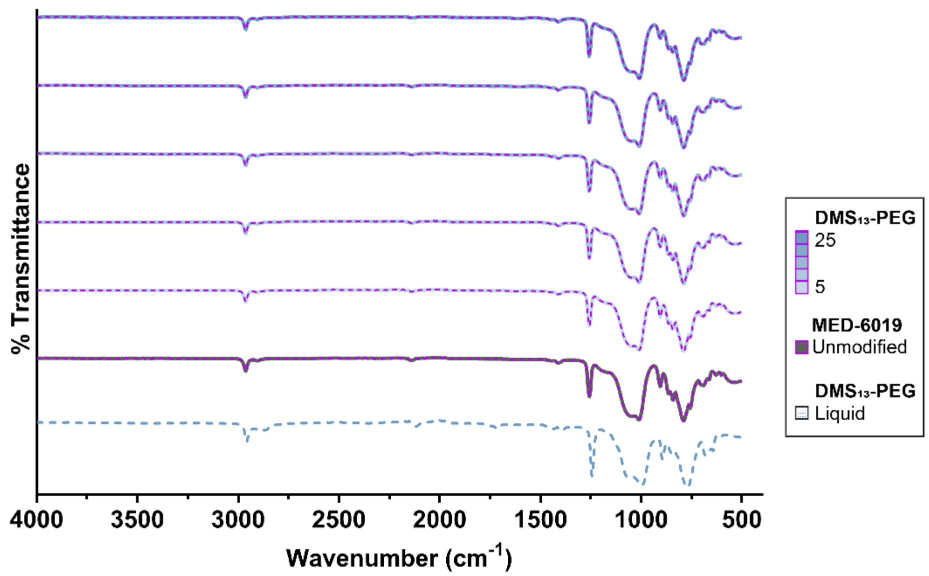

Figure S12. ATR-FTIR spectra of unmodified silicone (MED-6019; 0% Ph) and silicones modified with DMS_{13}-PEG at all concentrations (5 – 25 µmol g^{-1}). Color gradient (top of legend) refers to concentration of DMS_{13}-PEG (µmol g^{-1}) added to silicone.

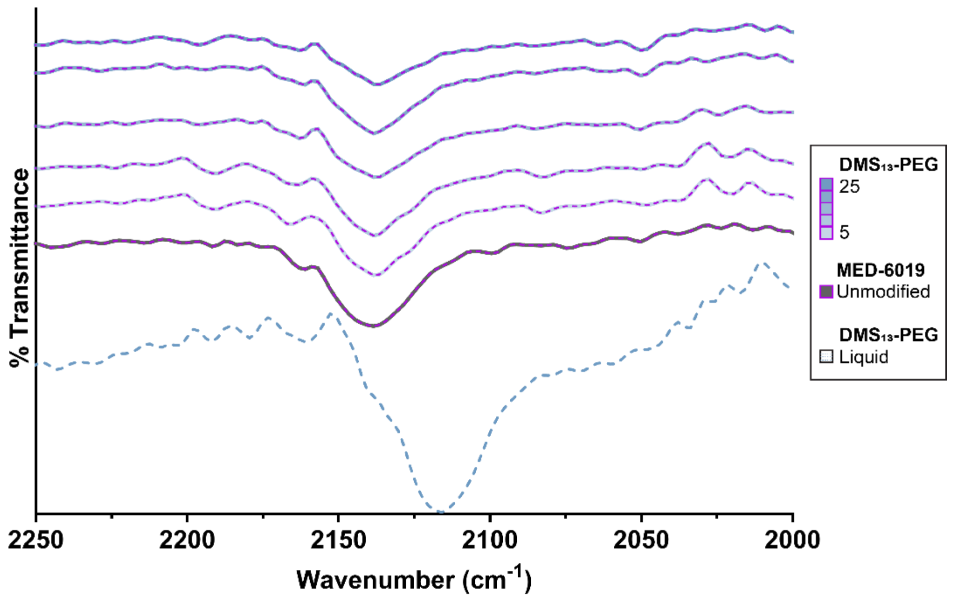

Figure S13. ATR-FTIR spectra of unmodified silicone (MED-6019; 0% Ph) and silicones modified with DMS_{13}-PEG at all concentrations (5 – 25 µmol g^{-1}). Color gradient (top of legend) refers to concentration of DMS_{13}-PEG (µmol g^{-1}) added to silicone. Characteristic Si-H peak at 2280-2080 cm^{-1}.

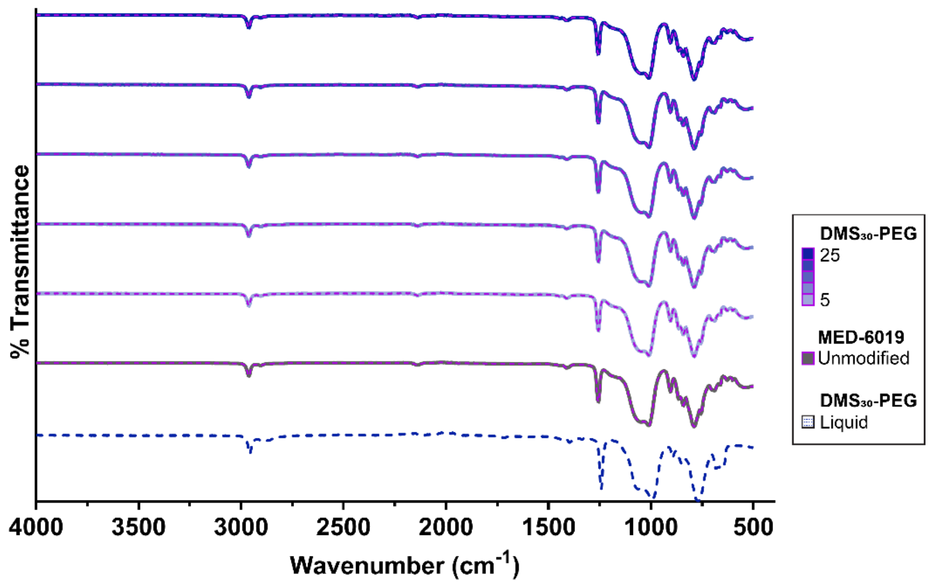

Figure S14. ATR-FTIR spectra of unmodified silicone (MED-6019; 0% Ph) and silicones modified with DMS_{30}-PEG at all concentrations (5 – 25 µmol g^{-1}). Color gradient (top of legend) refers to concentration of DMS_{30}-PEG (µmol g^{-1}) added to silicone.

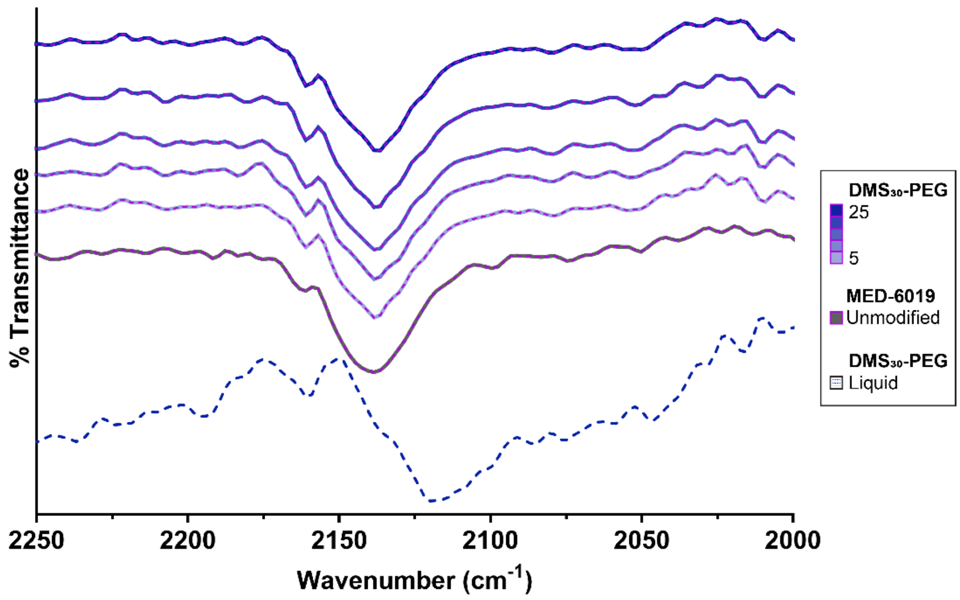

Figure S15. ATR-FTIR spectra of unmodified silicone (MED-6019; 0% Ph) and silicones modified with DMS_{30}-PEG at all concentrations (5 – 25 µmol g^{-1}). Color gradient (top of legend) refers to concentration of DMS_{30}-PEG (µmol g^{-1}) added to silicone. Characteristic Si-H peak at 2280-2080 cm^{-1}.

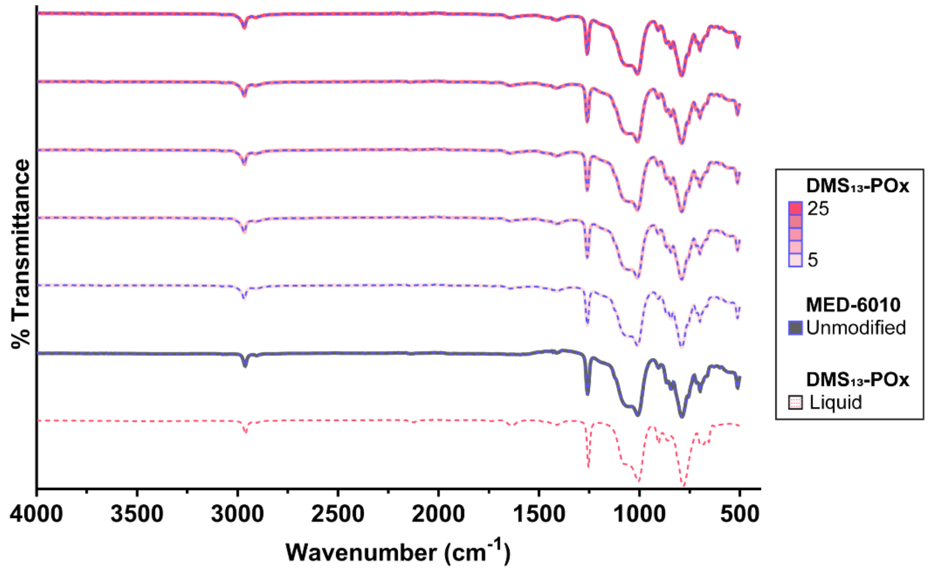

Figure S16. ATR-FTIR spectra of unmodified silicone (MED-6010; 5.8% Ph) and silicones modified with DMS_{13}-POx at all concentrations (5 – 25 µmol g^{-1}). Color gradient (top of legend) refers to concentration of DMS_{13}-POx (µmol g^{-1}) added to silicone.

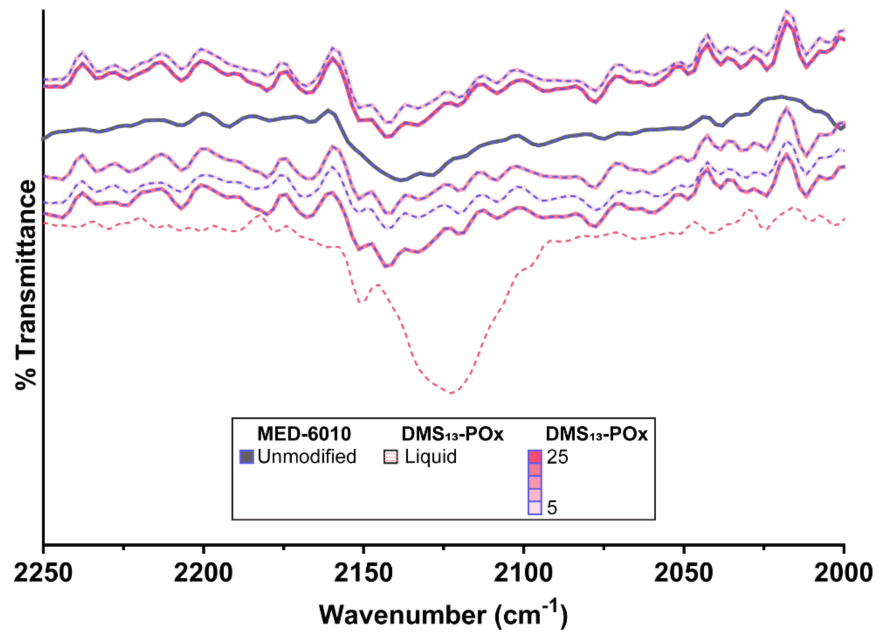

Figure S17. ATR-FTIR spectra of unmodified silicone (MED-6010; 5.8% Ph) and silicones modified with DMS_{13}-POx at all concentrations (5 – 25 µmol g^{-1}). Color gradient (right of legend) refers to concentration of DMS_{13}-POx (µmol g^{-1}) added to silicone. Characteristic Si-H peak at 2280-2080 cm^{-1}.

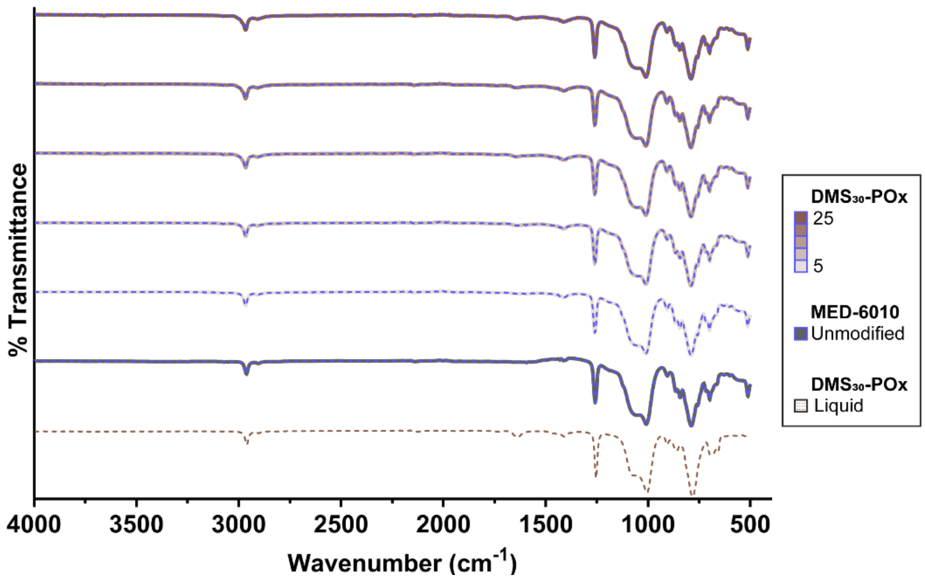

Figure S18. ATR-FTIR spectra of unmodified silicone (MED-6010; 5.8% Ph) and silicones modified with DMS_{30}-POx at all concentrations (5 – 25 µmol g^{-1}). Color gradient (top of legend) refers to concentration of DMS_{30}-POx (µmol g^{-1}) added to silicone.

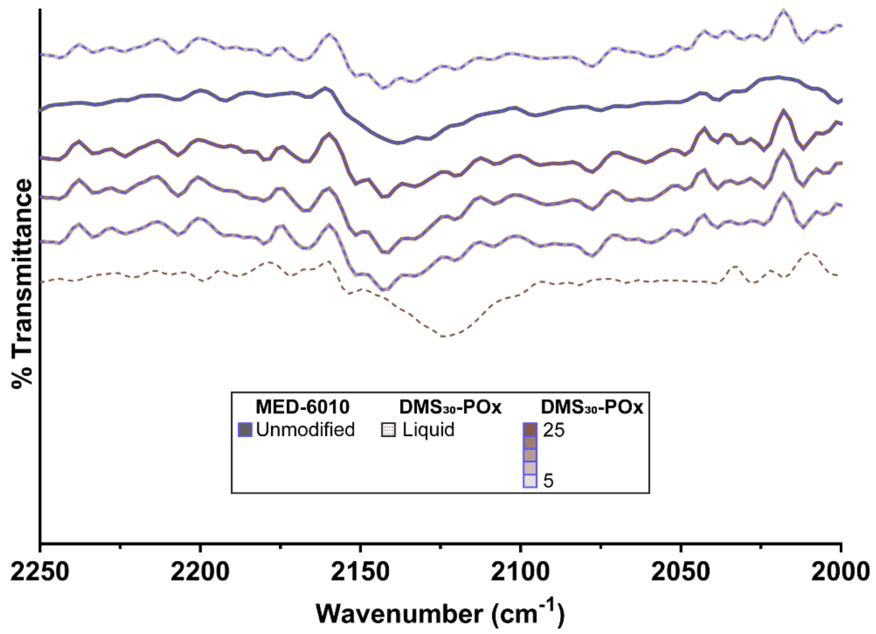

Figure S19. ATR-FTIR spectra of unmodified silicone (MED-6010; 5.8% Ph) and silicones modified with DMS_{30}-POx at all concentrations (5 – 25 µmol g^{-1}). Color gradient (right of legend) refers to concentration of DMS_{30}-POx (µmol g^{-1}) added to silicone. Characteristic Si-H peak at 2280-2080 cm^{-1}.

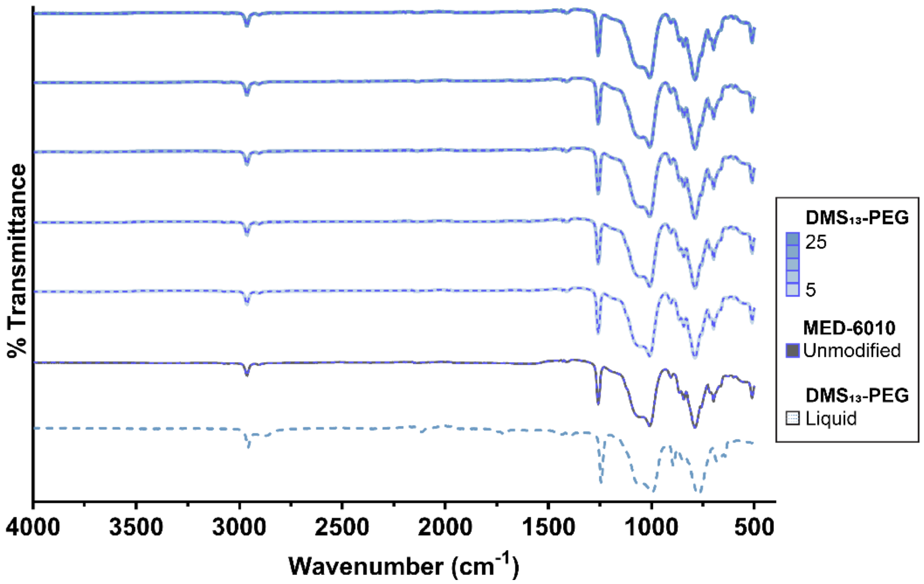

Figure S20. ATR-FTIR spectra of unmodified silicone (MED-6010; 5.8% Ph) and silicones modified with DMS_{13}-PEG at all concentrations (5 – 25 µmol g^{-1}). Color gradient (top of legend) refers to concentration of DMS_{13}-PEG (µmol g^{-1}) added to silicone.

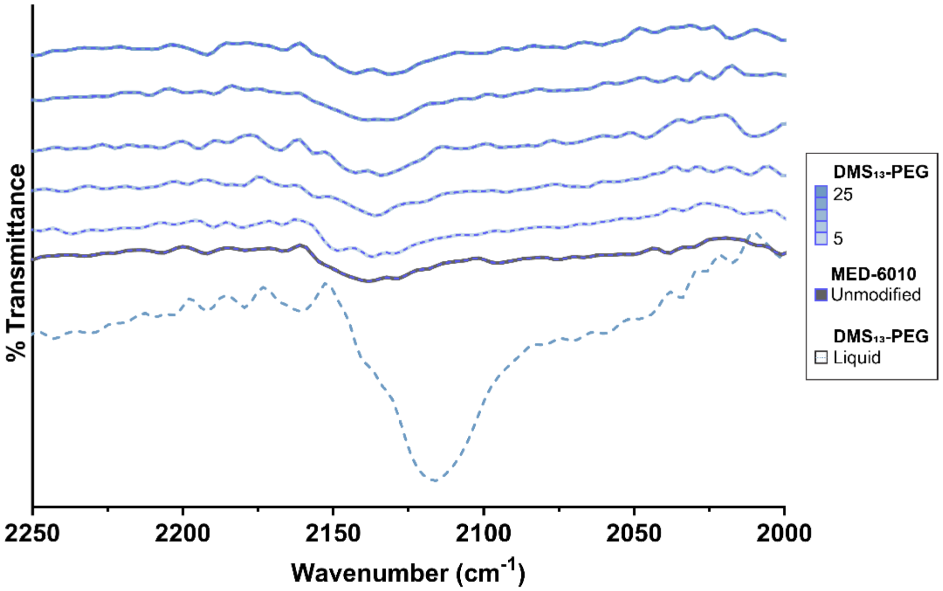

Figure S21. ATR-FTIR spectra of unmodified silicone (MED-6010; 5.8% Ph) and silicones modified with DMS_{13}-PEG at all concentrations (5 – 25 µmol g^{-1}). Color gradient (top of legend) refers to concentration of DMS_{13}-PEG (µmol g^{-1}) added to silicone. Characteristic Si-H peak at 2280-2080 cm^{-1}.

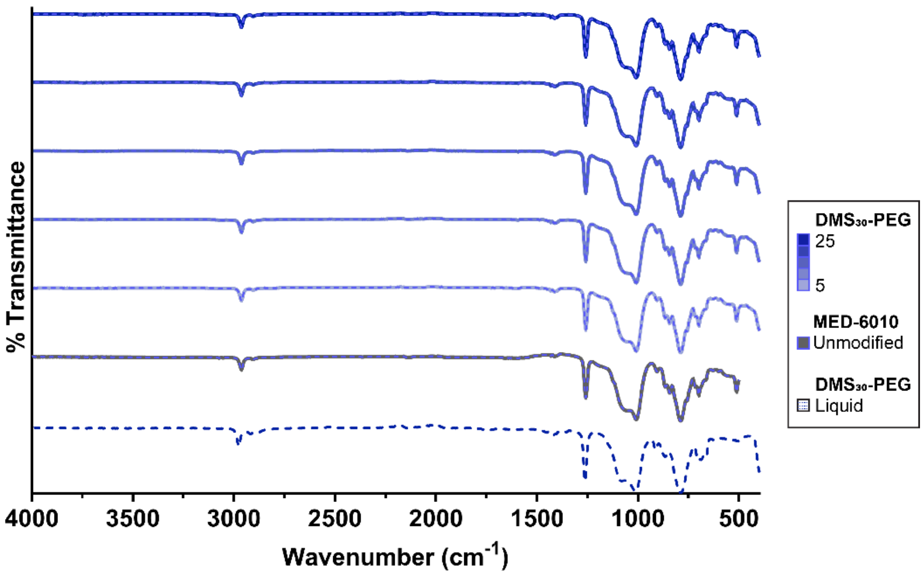

Figure S22. ATR-FTIR spectra of unmodified silicone (MED-6010; 5.8% Ph) and silicones modified with DMS_{30}-PEG at all concentrations (5 – 25 µmol g^{-1}). Color gradient (top of legend) refers to concentration of DMS_{30}-PEG (µmol g^{-1}) added to silicone.

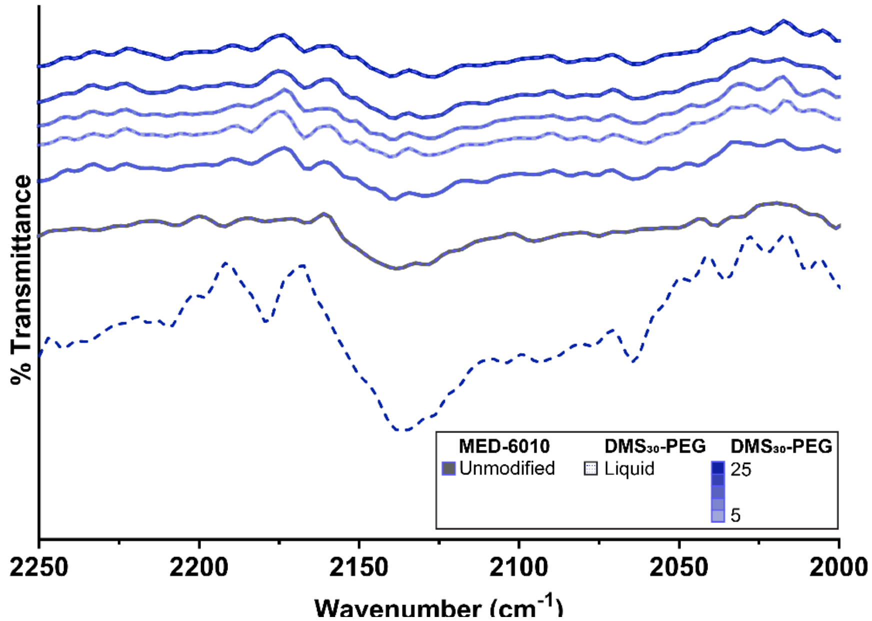

Figure S23. ATR-FTIR spectra of unmodified silicone (MED-6010; 5.8% Ph) and silicones modified with DMS_{30}-PEG at all concentrations (5 – 25 µmol g^{-1}). Color gradient (right of legend) refers to concentration of DMS_{30}-PEG (µmol g^{-1}) added to silicone. Characteristic Si-H peak at 2280-2080 cm^{-1}

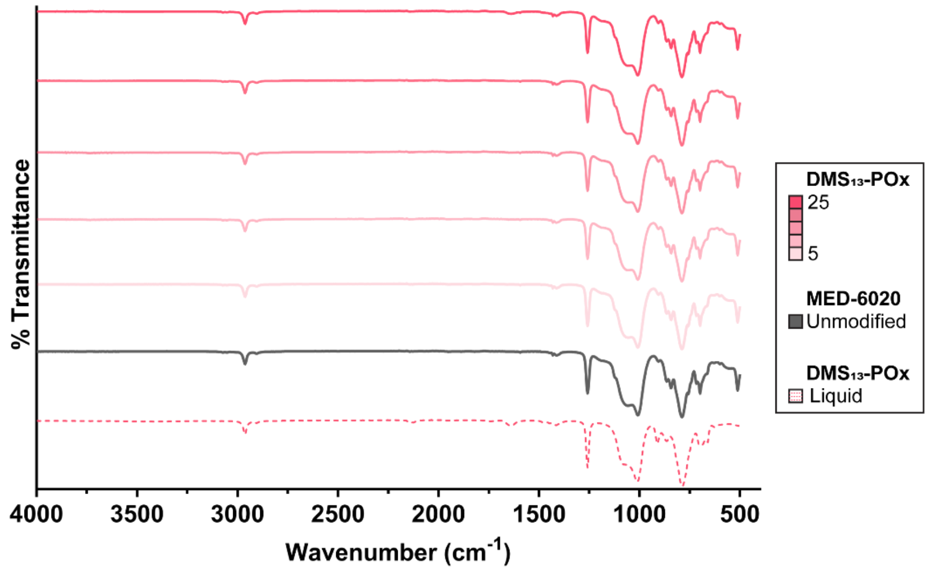

Figure S24. ATR-FTIR spectra of unmodified silicone (MED-6020; 8.5% Ph) and silicones modified with DMS_{30}-POx at all concentrations (5 – 25 µmol g^{-1}). Color gradient (top of legend) refers to concentration of DMS_{30}-POx (µmol g^{-1}) added to silicone.

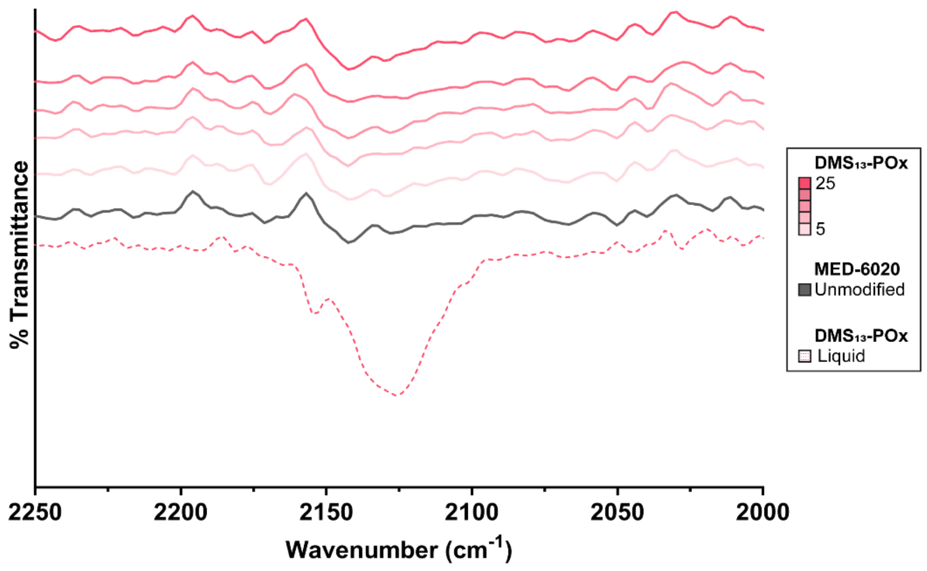

Figure S25. ATR-FTIR spectra of unmodified silicone (MED-6020; 8.5% Ph) and silicones modified with DMS_{13}-POx at all concentrations (5 – 25 µmol g^{-1}). Color gradient (top of legend) refers to concentration of DMS_{13}-POx (µmol g^{-1}) added to silicone. Characteristic Si-H peak at 2280-2080 cm^{-1}.

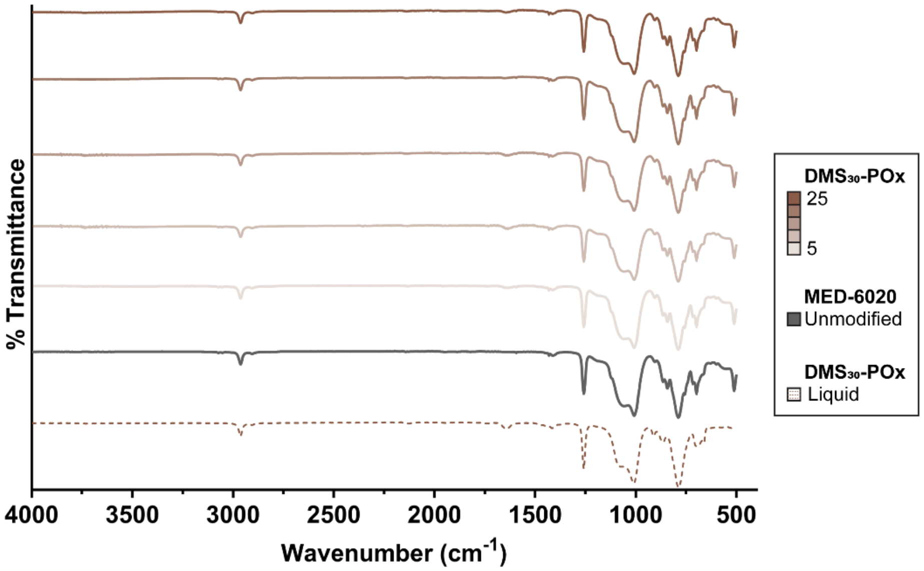

Figure S26. ATR-FTIR spectra of unmodified silicone (MED-6020; 8.5% Ph) and silicones modified with DMS_{30}-POx at all concentrations (5 – 25 µmol g^{-1}). Color gradient (top of legend) refers to concentration of DMS_{30}-POx (µmol g^{-1}) added to silicone.

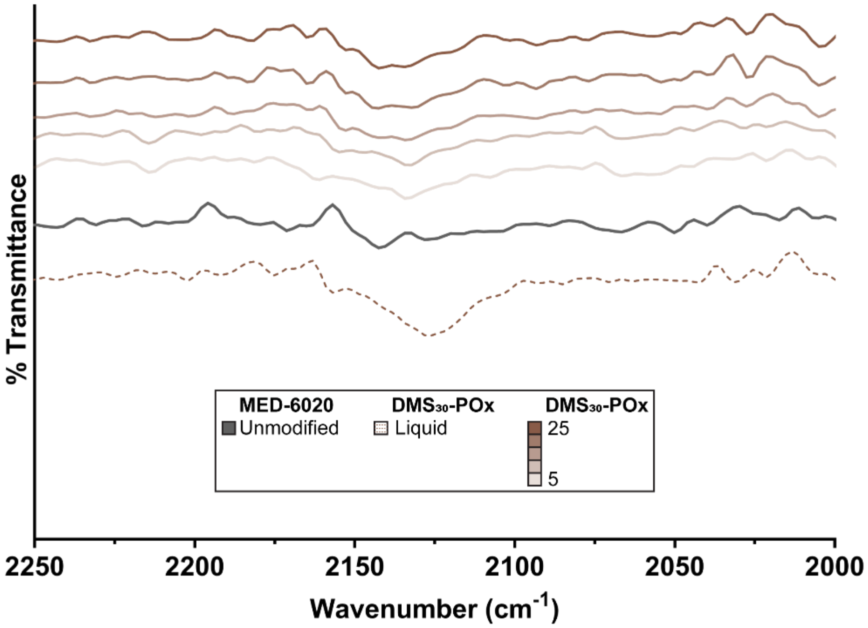

Figure S27. ATR-FTIR spectra of unmodified silicone (MED-6020; 8.5% Ph) and silicones modified with DMS_{30}-POx at all concentrations (5 – 25 µmol g^{-1}). Color gradient (right of legend) refers to concentration of DMS_{30}-POx (µmol g^{-1}) added to silicone. Characteristic Si-H peak at 2280-2080 cm^{-1}.

Note: ATR-FTIR spectra of unmodified silicone (MED-6020; 8.5% Ph) and silicones modified with DMS_{13}-PEG and DMS_{30}-PEG at all concentrations (5 – 25 µmol g^{-1}) reported by Marmo et al. [1]

Table S3. Aqueous-induced mass loss of unmodified silicones (MED-6019, -6010, and -6020) and silicones modified with POx- and PEG-based SMAs.

	Concentration (µmol g^{-1})	MED-6019 [0% Ph] Mass loss (%)	MED-6010 [5.8% Ph] Mass loss (%)	MED-6020 [8.5% Ph] Mass loss (%)
**Unmodified**	---	0.23 ± 0.14	-0.18 ± 0.35	0.02 ± 0. 08
**DMS_{13}-POx**				
	5	0.05 ± 0.12	0.40 ± 1.06	0.98 ± 0.67*
	10	-0.12 ± 0.16	0.18 ± 0.99	0.19 ± 0.43
	15	-0.07 ± 0.16	$-1.36e^{-03}$ ± 0.73	0.01 ± 0.24
	20	$-4.72e^{-03}$ ± 0.21	0.61 ± 0.82	0.36 ± 0.54
	25	0.04 ± 0.19	0.67 ± 0.95	0.57± 0.88
**DMS_{30}-POx**				
	5	0.06 ± 0.42	0.79 ± 1.78	0.41 ± 0.47
	10	0.09 ± 0.12	0.66 ± 0.70	0.06 ± 0.22
	15	0.13 ± 0.24	0.16 ± 0.64	0.29 ± 0.62
	20	0.36 ± 0.68	0.52 ± 0.29	0.06 ± 0.12
	25	0.11 ± 0.24	0.67 ± 1.25	0.51 ± 0.71
**DMS_{13}-PEG**				
	5	0.14 ± 0.32	$2.88e^{-03}$ ± 0.37	0.04 ± 0.08 ŧ
	10	-0.13 ± 0.18	-0.09 ± 0.47	0.10 ± 0.09 ŧ
	15	-0.08 ± 0.30	0.34 ± 0.38	0.13 ± 0.15 ŧ
	20	-0.04 ± 0.31	0.70 ± 0.66*	0.02 ± 0.09 ŧ
	25	0.15 ± 0.29	0.87 ± 0.40*	0.09 ± 0.11 ŧ
**DMS_{30}-PEG**				
	5	0.08 ± 0.18	0.26 ± 0.17	0.03 ± 0.10 ŧ
	10	-0.07 ± 0.31	0.07 ± 0.24	0.14 ± 0.14 ŧ
	15	0.20 ± 0.45	0.16 ± 0.34	0.20 ± 0.13 ŧ
	20	0.00 ± 0.00	-0.10 ± 0.53	0.28 ± 0.16* ŧ
	25	-0.17 ± 0.55	-0.24 ± 0.14	0.41 ± 0.19* ŧ

ŧ Data reported by Marmo et al. [1]. * $p < 0.05$ vs corresponding unmodified silicone.

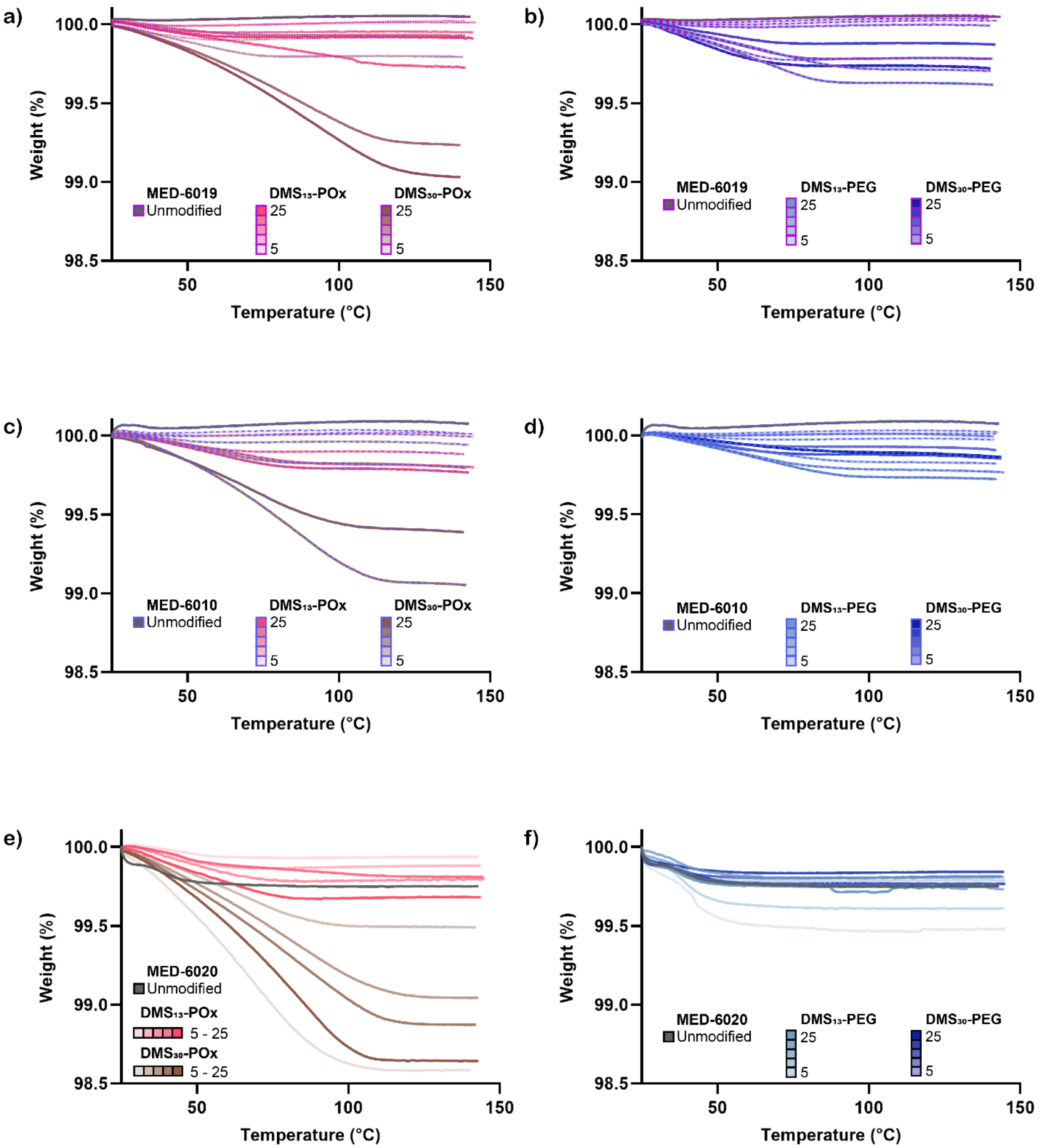

Figure S28. TGA of unmodified and SMA-modified silicones: **(a, b)** MED-6019 [0% Ph], **(c, d)** MED-6010 [5.8% Ph], and **(e, f)** MED-6020 [8.5% Ph] each with DMS_{13}-POx, DMS_{13}-PEG, DMS_{30}-POx, and DMS_{30}-PEG (5 – 25 µmol g^{-1}).

Table S4. Water uptake of unmodified silicones (MED-6019, -6010, and -6020) and silicones modified with POx- and PEG-based SMAs.

	Concentration (μmol g^{-1})	MED-6019 [0% Ph] Water uptake (%)	MED-6010 [5.8% Ph] Water uptake (%)	MED-6020 [8.5% Ph] Water uptake (%)
**Unmodified**	---	0.02 ± 2.70e^{-03}	0.02 ± 0.01	0.02 ± 0.02
**DMS$_{13}$-POx**				
	5	0.03 ± 0.01	0.01 ± 0.01	0.01 ± 0.01
	10	0.02 ± 0.01	0.01 ± 2.69e^{-03}	0.01 ± 0.01
	15	2.47e^{-03} ± 1.96e^{-03}	0.03 ± 0.01	0.03 ± 0.04
	20	0.02 ± 0.02	0.10 ± 0.01	0.01 ± 2.81e^{-03}
	25	0.01± 4.27e^{-03}	0.12 ± 0.03	0.11 ± 0.07
**DMS$_{30}$-POx**				
	5	0.01 ± 0.01	4.47e^{-03} ± 8.62e^{-04}	0.80 ± 0.18*
	10	0.06 ± 0.04	0.01 ± 1.86e^{-03}	0.24 ± 0.08
	15	0.02 ± 0.02	0.10 ± 0.04	0.63 ± 0.05*
	20	0.56 ± 0.06*	0.68 ± 0.21	0.74 ± 0.05*
	25	0.73 ± 0.02*	0.39 ± 0.14	3.95 ± 0.50*
**DMS$_{13}$-PEG**				
	5	0.03 ± 4.97e^{-03}	0.01 ± 0.01	0.06 ± 0.03 ᵻ
	10	0.03 ± 2.31e^{-03}	0.01 ± 3.99e^{-03}	0.03 ± 0.04 ᵻ
	15	0.05 ± 0.03	0.09 ± 0.01	0.01 ± 0.01 ᵻ
	20	0.17 ± 0.03*	0.11 ± 0.05*	0.01 ± 0.01 ᵻ
	25	0.19 ± 0.04*	0.15 ± 0.03*	0.01 ± 0.01 ᵻ
**DMS$_{30}$-PEG**				
	5	0.02 ± 2.96e^{-03}	0.01 ± 4.62e^{-03}	0.01 ± 0.01 ᵻ
	10	0.01 ± 0.01	0.01 ± 2.85e^{-03}	0.02 ± 0.02 ᵻ
	15	0.09 ± 0.02*	0.03 ± 0.02	0.00 ± 0.00 ᵻ
	20	0.04 ± 0.02	0.05 ± 0.03	0.00 ± 0.00 ᵻ
	25	0.07 ± 0.07	0.07 ± 0.06	0.01 ± 0.01 ᵻ

ᵻ Data reported by Marmo et al. [1]. * $p < 0.05$ vs corresponding unmodified silicone.

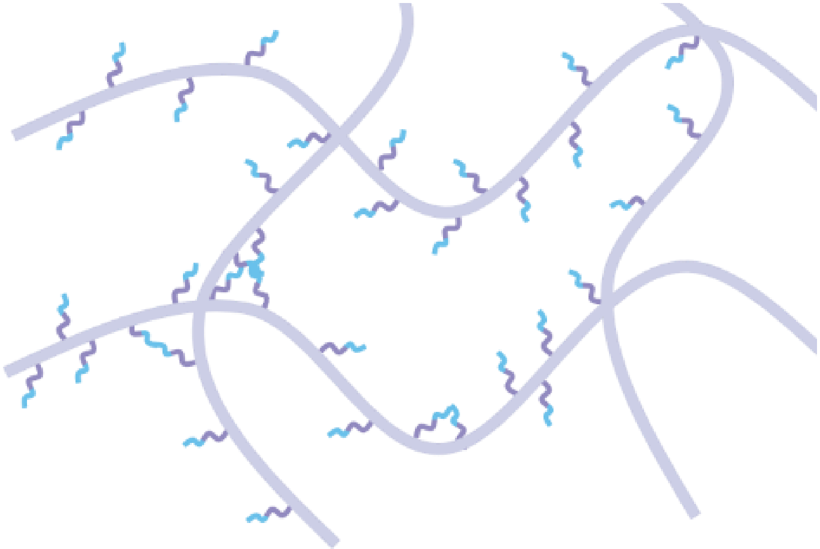

Figure S29. Depiction of POx- and PEG-silane amphiphiles within a cured silicone, with PEtOx and PEG remaining as dangling free ends.

Table S5. Tensile modulus of unmodified silicones (MED-6019, -6010, and -6020) and silicones modified with POx- and PEG-based SMAs.

	Concentration (μmol g^{-1})	MED-6019 [0% Ph] Modulus (MPa)	MED-6010 [5.8% Ph] Modulus (MPa)	MED-6020 [8.5%] Modulus (MPa)
**Unmodified**	---	8.81 ± 0.90	1.71 ± 0.39	0.94 ± 0.06
**DMS_{13}-POx**				
	5	6.97 ± 0.30	2.12 ± 0.09	0.62 ± 0.16
	10	5.62 ± 0.21	1.65 ± 0.23	0.62 ± 0.28
	15	4.79 ± 0.14	1.26 ± 0.10	0.70 ± 0.15
	20	4.86 ± 0.85	1.14 ± 0.10	0.67 ± 0.09
	25	4.78 ± 0.56	1.09 ± 0.13	0.71 ± 0.20
**DMS_{30}-POx**				
	5	6.67 ± 0.27	1.15 ± 0.09	0.57 ± 0.13
	10	5.60 ± 0.71	1.24 ± 0.34	0.39 ± 0.15
	15	4.88± 0.13	0.85 ± 0.26	0.53 ± 0.08
	20	3.90 ± 0.21	0.93 ± 0.04	0.46 ± 0.01
	25	2.43 ± 0.08	0.77 ± 0.11	0.30 ± 0.08
**DMS_{13}-PEG**				
	5	7.25 ± 0.99	1.69 ± 0.12	1.08 ± 0.12 ŧ
	10	7.53 ± 0.85	1.44 ± 0.10	1.12 ± 0.05 ŧ
	15	6.22 ± 0.66	1.29 ± 0.17	1.04 ± 0.06 ŧ
	20	6.86 ± 1.26	1.37 ± 0.17	0.97 ± 0.04 ŧ
	25	7.10 ± 0.66	1.35 ± 0.13	1.07 ± 0.04 ŧ
**DMS_{30}-PEG**				
	5	8.40 ± 0.76	1.46 ± 0.04	0.90 ± 0.06 ŧ
	10	7.02 ± 0.38	1.43 ± 0.14	1.03 ± 0.04 ŧ
	15	5.44 ± 0.59	1.33 ± 0.11	0.94 ± 0.04 ŧ
	20	5.61 ± 0.16	1.21 ± 0.14	0.84 ± 0.06 ŧ
	25	5.51 ± 0.12	1.43 ± 0.09	0.89 ± 0.16 ŧ

ŧ Data reported by Marmo et al. [1]

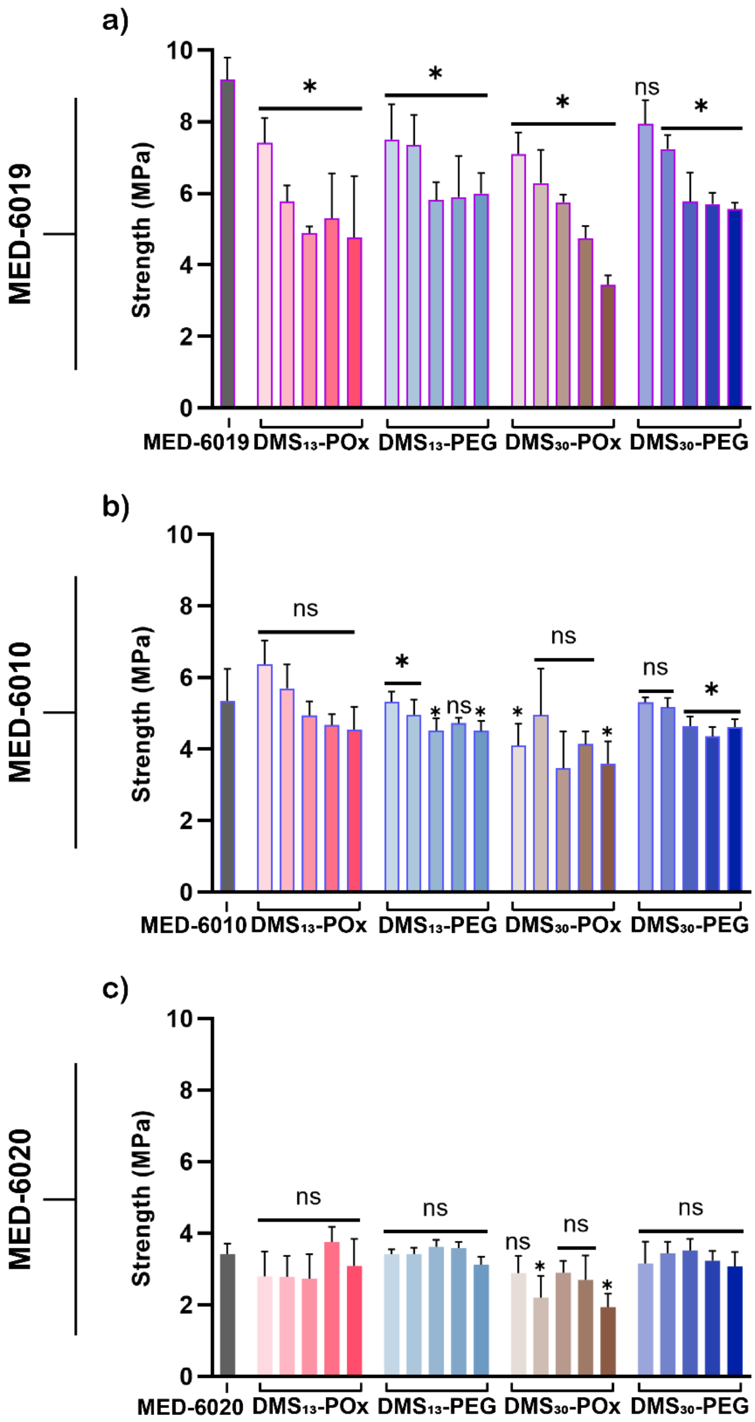

Figure S30. Tensile strength of silicones **(a)** MED-6019 [0% Ph], **(b)** MED-6010 [5.8% Ph], and **(c)** MED-6020 [8.5% Ph] unmodified and modified with POx- and PEG-based SMAs. * $p < 0.05$ vs corresponding unmodified silicone. DMS_{13}-PEG and DMS_{30}-PEG in MED-6020 data reported by Marmo et al. [1]

Table S6. Tensile strength of unmodified silicones (MED-6019, -6010, and -6020) and silicones modified with POx- and PEG-based SMAs.

	**Concentration (µmol g^{-1})**	**MED-6019 [0% Ph] Strength (MPa)**	**MED-6010 [5.8% Ph] Strength (MPa)**	**MED-6020 [8.5% Ph] Strength (MPa)**
**Unmodified**	---	9.18 ± 0.61	5.35 ± 0.89	3.42 ± 0.13
**DMS_{13}-POx**				
	5	7.41 ± 0.69	6.38 ± 0.66	2.80 ± 0.69
	10	5.78 ± 0.44	5.71 ± 0.67	2.78 ± 0.59
	15	4.89 ± 0.19	4.94 ± 0.38	2.74 ± 0.69
	20	5.30 ± 1.26	4.67 ± 0.31	3.76 ± 0.41
	25	4.76 ± 1.72	4.55 ± 0.63	3.09 ± 0.76
**DMS_{30}-POx**				
	5	7.10 ± 0.59	4.11 ± 0.60	2.89 ± 0.48
	10	6.28 ± 0.93	4.96 ± 1.30	2.21 ± 0.61
	15	5.75 ± 0.21	3.47 ± 1.02	2.90 ± 0.33
	20	4.75 ± 0.34	4.14 ± 0.35	2.70 ± 0.68
	25	3.45 ± 0.27	3.60 ± 0.61	1.93 ± 0.38
**DMS_{13}-PEG**				
	5	7.51 ± 0.98	5.33 ± 0.29	3.42 ± 0.13 ᵻ
	10	7.34 ± 0.85	4.96 ± 0.42	3.42 ± 0.18 ᵻ
	15	5.81 ± 0.50	4.52 ± 0.34	3.62 ± 0.20 ᵻ
	20	5.91 ± 1.14	4.72 ± 0.16	3.60 ± 0.15 ᵻ
	25	6.01 ± 0.57	4.50 ± 0.29	3.13 ± 0.22 ᵻ
**DMS_{30}-PEG**				
	5	7.94 ± 0.66	5.30 ± 0.15	3.17 ± 0.61 ᵻ
	10	7.24 ± 0.40	5.17 ± 0.26	3.45 ± 0.31 ᵻ
	15	5.78 ± 0.80	4.65 ± 0.26	3.52 ± 0.32 ᵻ
	20	5.71 ± 0.31	4.35 ± 0.27	3.23 ± 0.28 ᵻ
	25	5.56 ± 0.17	4.62 ± 0.22	3.08 ± 0.40 ᵻ

ᵻ Data reported by Marmo et al. [1]

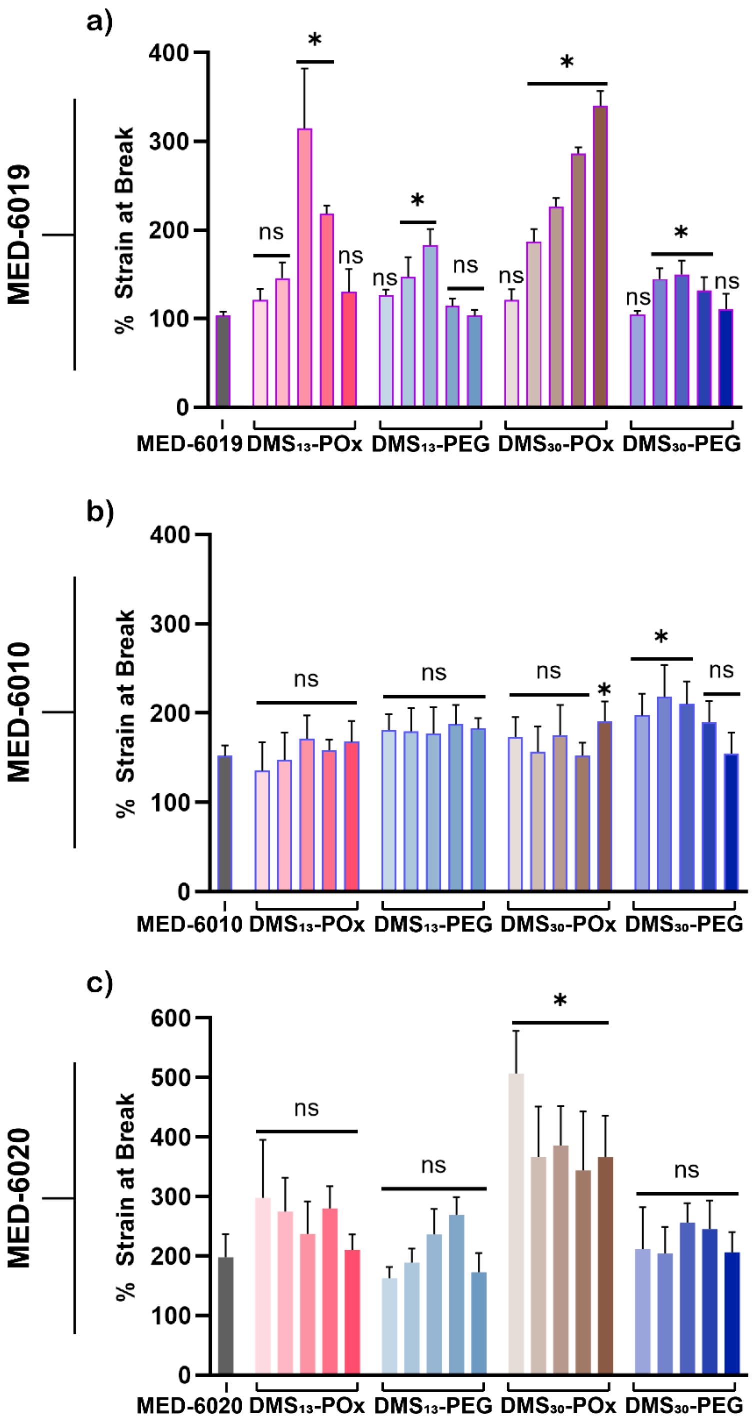

Figure S31. % Strain at break (%ε) of silicones **(a)** MED-6019 [0% Ph], **(b)** MED-6010 [5.8% Ph], and **(c)** MED-6020 [8.5% Ph] unmodified and modified with POx- and PEG-based SMAs. * $p < 0.05$ vs corresponding unmodified silicone. DMS_{13}-PEG and DMS_{30}-PEG in MED-6020 data reported by Marmo et al. [1]

Table S7. Strain at break (%ε) of unmodified silicones (MED-6019, -6010, and -6020) and silicones modified with POx- and PEG-based SMAs.

	Concentration (μmol g^{-1})	MED-6019 [0% Ph] (%ε)	MED-6010 [5.8% Ph] (%ε)	MED-6020 [8.5% Ph] (%ε)
**Unmodified**	---	103.5 ± 4.7	152.1 ± 11.9	198.34 ± 38.72
**DMS_{13}-POx**				
	5	120.7 ± 12.8	135.6 ± 31.8	298.5 ± 96.6
	10	146.1 ± 17.3	147.9 ± 30.3	274.9 ± 56.8
	15	314.7 ± 67.6	171.4 ± 26.0	237.4 ± 54.6
	20	218.5 ± 9.1	158.6 ± 11.7	280.4 ± 36.7
	25	130.3 ± 25.9	168.1 ± 23.0	210.7 ± 26.1
**DMS_{30}-POx**				
	5	121.0 ± 12.1	173.1 ± 22.7	507.0 ± 71.8
	10	186.3± 14.9	157.0 ± 28.5	367.1 ± 84.0
	15	226.4 ± 9.8	175.3 ± 33.8	386.1 ± 65.4
	20	286.0 ± 7.4	152.4 ± 14.8	343.7 ± 99.3
	25	340.2 ± 16.6	191.2 ± 22.0	366.8 ± 68.8
**DMS_{13}-PEG**				
	5	126.6 ± 5.9	181.1 ± 17.8	162.8 ± 19.2 Ŧ
	10	147.1 ± 21.9	179.7 ± 25.9	189.2 ± 23.6 Ŧ
	15	182.4 ± 18.7	177.3 ± 29.7	236.6 ± 42.8 Ŧ
	20	114.0 ± 8.8	187.4 ± 21.7	269.5 ± 29.9 Ŧ
	25	103.6 ± 6.6	182.7 ± 11.7	173.4 ± 32.0 Ŧ
**DMS_{30}-PEG**				
	5	104.9 ± 3.9	197.6 ± 24.1	211.5 ± 70.9 Ŧ
	10	144.8 ± 12.0	218.6 ± 35.5	204.7 ± 44.4 Ŧ
	15	150.2 ± 15.2	210.1 ± 25.7	256.5 ± 32.5 Ŧ
	20	131.6 ± 15.6	189.7 ± 23.8	245.8 ± 48.0 Ŧ
	25	110.9 ± 16.9	155.3 ± 22.8	206.0 ± 34.6 Ŧ

Ŧ Data reported by Marmo et al. [1]

Table S8. Static contact angle (DI water) (θ_{static}) values for unmodified silicone (MED-6019; 0% Ph) and silicone*s* modified with POx- and PEG-based SMAs.

	Concentration (µmol g^{-1})	t = 0 min	t = 1 min	t = 2 min	t = 3 min
**MED-6019**	---	106.3 ± 2.7	105.6 ± 1.3	106.4 ± 3.8	104.5 ± 1.4
**DMS$_{13}$-POx**					
	5	96.9 ± 4.5	95.5 ± 4.6	94.1 ± 4.6	92.8 ± 4.5
	10	99.4 ± 3.5	98.0 ± 3.6	96.7 ± 3.6	95.4 ± 3.7
	15	96.4 ± 3.1	94.4 ± 3.0	92.6 ± 2.8	91.4 ± 2.7
	20	101.5 ± 2.8	94.9 ± 3.5	93.6 ± 3.5	91.6 ± 2.6
	25	104.0 ± 7.6	95.9 ± 2.5	94.9 ± 2.6	93.7 ± 2.6
**DMS$_{30}$-POx**					
	5	98.2 ± 3.9	96.5 ± 1.9	95.4 ± 1.9	94.3 ± 1.8
	10	95.2 ± 1.1	93.6 ± 1.0	92.5 ± 1.0	91.4 ± 1.1
	15	99.4 ± 3.0	97.1 ± 2.9	95.0 ± 1.6	94.3 ± 1.6
	20	97.8± 5.2	95.7 ± 3.8	94.1 ± 3.4	93.6 ± 3.5
	25	103.9 ± 3.6	100.8 ± 0.9	99.1 ± 3.2	98.4 ± 3.0
**DMS$_{13}$-PEG**					
	5	113.6 ± 6.3	107.8 ± 1.8	107.1 ± 1.9	106.3 ± 1.9
	10	115.5 ± 4.5	107.2 ± 2.0	106.5 ± 2.1	105.9 ± 2.1
	15	98.5 ± 1.7	92.9 ± 1.3	90.9 ± 1.6	87.0 ± 2.7
	20	107.0 ± 4.6	102.3 ± 3.3	94.1 ± 7.1	83.0 ± 5.8
	25	102.8 ± 3.3	100.4 ± 1.4	91.8 ± 8.3	74.5 ± 8.2
**DMS$_{30}$-PEG**					
	5	115.0 ± 3.1	104.9 ± 1.2	104.2 ± 1.2	103.5 ± 1.2
	10	113.5 ± 6.1	105.0 ± 2.4	104.3 ± 2.4	103.8 ± 2.3
	15	111.2 ± 4.9	102.7 ± 4.3	98.4 ± 4.9	89.9 ± 9.0
	20	109.8 ± 3.7	102.7 ± 0.8	97.3 ± 2.7	87.4 ± 6.3
	25	105.8 ± 5.6	94.0 ± 4.9	76.6 ± 7.1	62.8 ± 10.1

Table S9. Static contact angle (DI water) (θ_{static}) values for unmodified silicone (MED-6010; 5.8% Ph) and silicone*s* modified with POx- and PEG-based SMAs.

	Concentration (μmol g^{-1})	t = 0 min	t = 1 min	t = 2 min	t = 3 min
**MED-6010**	---	116.5 ± 6.9	113.3 ± 4.8	112.1 ± 4.9	111.0 ± 4.8
**DMS_{13}-POx**					
	5	116.6 ± 5.2	114.7 ± 4.5	112.0 ± 4.1	111.0 ± 3.6
	10	65.6 ± 10.2	48.9 ± 16.0	48.1 ± 16.0	46.4 ± 13.2
	15	79.2 ± 5.0	72.6 ± 2.8	71.4 ± 2.3	69.9 ± 2.6
	20	64.1 ± 9.6	49.7 ± 23.1	40.2 ± 21.3	30.9 ± 14.7
	25	78.5 ± 8.1	61.8 ± 21.7	53.8 ± 19.7	27.2 ± 5.1
**DMS_{30}-POx**					
	5	122.8 ± 2.0	114.0 ± 3.8	105.3 ± 3.0	103.4 ± 2.6
	10	106.2 ± 5.8	100.2 ± 6.8	97.2 ± 6.3	95.7 ± 8.6
	15	96.9 ± 8.5	96.2 ± 5.6	94.8 ± 5.6	93.4 ± 5.6
	20	85.2 ± 15.0	70.3 ± 20.1	61.3 ± 22.2	54.7 ± 21.5
	25	78.6 ± 6.6	76.8 ± 6.4	75.3 ± 6.4	73.6 ± 6.3$
**DMS_{13}-PEG**					
	5	106.3 ± 7.1	75.6 ± 10.0	44.6 ± 4.9	35.4 ± 3.5
	10	93.1 ± 4.6	23.2 ± 4.9	20.8 ± 5.0	19.9 ± 4.8
	15	40.7 ± 12.2	17.6 ± 3.1	15.6 ± 4.9	13.8 ± 5.8 ┐
	20	27.3 ± 3.8	17.6 ± 3.1	17.1 ± 3.4	16.6 ± 3.5 │ ns
	25	30.2 ± 5.0	20.3 ± 3.8	18.4 ± 3.7	18.1 ± 4.7 ┘
**DMS_{30}-PEG**					
	5	116.7 ± 2.5	113.5 ± 3.0	104.5 ± 2.8	99.0 ± 4.5
	10	113.6 ± 2.0	95.6 ± 4.2	74.9 ± 3.0	56.7 ± 5.3
	15	105.9 ± 14.3	54.5 ± 7.6	26.4 ± 7.8	20.0 ± 3.3
	20	107.4 ± 12.6	29.7 ± 11.1	20.9 ± 4.7	16.6 ± 2.8
	25	98.3 ± 11.1	18.9 ± 1.9	16.3 ± 2.9	15.6 ± 4.0

$ $p < 0.05$ vs previous composition.

Table S10. Static contact angle (DI water) (θ_{static}) values for unmodified silicone (MED-6020; 8.5% Ph) and silicones modified with POx- and PEG-based SMAs.

	Concentration (µmol g^{-1})	t = 0 min	t = 1 min	t = 2 min	t = 3 min
**MED-6020**	---	112.9 ± 5.3	111.3 ± 4.1	110.6 ± 3.9	109.9 ± 3.8
**DMS$_{13}$-POx**					
	5	112.0 ± 2.8	110.2 ± 2.9	109.3 ± 2.8	105.8 ± 3.2
	10	96.2 ± 21.8	93.5 ± 18.5	88.3 ± 15.7	86.9 ± 15.5
	15	110.6 ± 8.8	99.9 ± 11.6	93.2 ± 9.2	90.2 ± 9.1
	20	59.5 ± 30.5	40.7 ± 6.8	38.8 ± 6.3	30.0 ± 10.7
	25	80.4 ± 39.7	27.4 ± 12.8	21.4 ± 6.1	19.3 ± 5.4
**DMS$_{30}$-POx**					
	5	107.1 ± 7.1	98.1 ± 9.1	96.9 ± 9.1	95.6 ± 9.0
	10	113.5 ± 9.4	104.9 ± 5.4	100.5 ± 6.3	96.4 ± 3.8
	15	94.1 ± 23.0	86.0 ± 15.0	85.3 ± 15.1	84.3 ± 14.9
	20	106.3 ± 16.4	99.1 ± 12.0	94.2 ± 8.8	92.9 ± 8.1
	25	80.2 ± 2.5	79.3 ± 2.2	78.5 ± 2.3	77.8 ± 2.3
**DMS$_{13}$-PEG**					
	5	114.7 ± 4.1 ꝥ	113.6 ± 3.8 ꝥ	112.6 ± 3.7 ꝥ	109.0 ± 7.2 ꝥ
	10	117.5 ± 4.6 ꝥ	115.8 ± 5.0 ꝥ	89.1 ± 11.3 ꝥ	71.7 ± 12.3 ꝥ
	15	112.0 ± 9.1 ꝥ	103.8 ± 8.2 ꝥ	69.9 ± 6.6 ꝥ	54.7 ± 4.1 ꝥ
	20	85.6 ± 4.4 ꝥ	31.5 ± 4.7 ꝥ	21.9 ± 4.1 ꝥ	15.8 ± 4.3 ꝥ
	25	62.2 ± 33.3 ꝥ	17.4 ± 3.6 ꝥ	13.9 ± 3.8 ꝥ	13.0 ± 3.3 ꝥ
**DMS$_{30}$-PEG**					
	5	108.8 ± 6.7 ꝥ	99.0 ± 7.4 ꝥ	57.9 ± 13.2 ꝥ	40.8 ± 9.9 ꝥ
	10	110.2 ± 5.8 ꝥ	59.2 ± 21.2 ꝥ	31.0 ± 11.9 ꝥ	21.6 ± 8.4 ꝥ
	15	117.1 ± 5.4 ꝥ	59.2 ± 9.6 ꝥ	35.4 ± 5.9 ꝥ	24.5 ± 4.7 ꝥ
	20	113.5 ± 8.1 ꝥ	32.3 ± 5.7 ꝥ	16.8 ± 4.1 ꝥ	11.8 ± 2.6 ꝥ
	25	85.3 ± 23.6 ꝥ	14.1 ± 4.2 ꝥ	11.3 ± 1.8 ꝥ	10.7 ± 1.0 ꝥ

ꝥ Data reported by Marmo et al. [1]

Table S11. Fibrinogen adsorption on unmodified silicones (MED-6019, -6010, and -6020) and silicones modified with POx- and PEG-based SMAs.

	**Concentration (μmol g^{-1})**	**Fibrinogen adsorption (ng cm^{-2})**
**MED-6019 (0% Ph)**	---	175.3 ± 20.0
**DMS_{13}-POx**	25	29.8 ± 10.9
**DMS_{30}-POx**	25	42.5 ± 4.3
**DMS_{13}-PEG**	25	16.8 ± 3.17
**DMS_{30}-PEG**	25	31.58 ± 12.8
**MED-6010 (0% Ph)**	---	150.2 ± 12.8
**DMS_{13}-POx**	25	121.1 ± 8.0
**DMS_{30}-POx**	25	52.8 ± 3.3
**DMS_{13}-PEG**	25	70.9 ± 7.0
**DMS_{30}-PEG**	25	58.0 ± 9.5
**MED-6020 (0% Ph)**	---	145.8 ± 12.5
**DMS_{13}-POx**	25	126.6 ± 5.9
**DMS_{30}-POx**	25	147.1 ± 21.9
**DMS_{13}-PEG**	25	30.9 ± 3.5
**DMS_{30}-PEG**	25	20.3 ± 4.1

Table S12. Crystal violet absorbance values of biofilm growth on unmodified silicones (MED-6019, -6010, and -6020) and silicones modified with POx- and PEG-based SMAs.

	Concentration (μmol g^{-1})	*C. albicans*	*E. coli*	*P. aeruginosa*	*S. aureus*	*S. epidermis*
**MED-6019**	---	0.756 ± 0.089	1.161 ± 0.124	0.474 ± 0.090	0.467 ± 0.039	0.835 ± 0.043
**DMS_{13}-POx**	25	0.010 ± 0.016	0.068 ± 0.061	0.128 ± 0.168	0.003 ± 0.049	0.077 ± 0.207
**DMS_{30}-POx**	25	0.019 ± 0.017	0.046 ± 0.029	0.240 ± 0.022	0.026 ± 0.004	0.014 ± 0.025
**DMS_{13}-PEG**	25	0.006 ± 0.023	0.010 ± 0.013	0.682 ± 0.049	0.0138 ± 0.007	0.005 ± 0.003
**DMS_{30}-PEG**	25	0.000 ± 0.001	0.008 ± 0.008	0.060 ± 0.078	0.027 ± 0.014	0.020 ± 0.016
**MED-6010**	---	0.879 ± 0.028	1.289 ± 0.136	0.625 ± 0.016	0.459 ± 0.008	0.907 ± 0.046
**DMS_{13}-POx**	25	0.176 ± 0.103	0.827 ± 0.083	0.629 ± 0.088	0.4407 ± 0.061	0.564 ± 0.041
**DMS_{30}-POx**	25	0.336 ± 0.063	0.320 ±0.166	0.572 ± 0.022	0.117 ± 0.049	0.020 ± 0.017
**DMS_{13}-PEG**	25	0.010 ± 0.008	0.018 ± 0.008	0.447 ± 0.012	0.018 ± 0.004	0.005 ± 0.001
**DMS_{30}-PEG**	25	0.007 ± 0.003	0.034 ± 0.013	0.329 ±0.211	0.035 ± 0.022	0.007 ± 0.006
**MED-6020**	---	1.039 ± 0.134	1.197 ± 0.020	0.535 ± 0.082	0.502 ± 0.055	0.024 ± 0.029
**DMS_{13}-POx**	25	0.108 ± 0.007	0.050 ± 0.006	0.276 ± 0.053	0.079 ± 0.026	0.031 ± 0.034
**DMS_{30}-POx**	25	0.029 ± 0.023	0.030 ± 0.020	0.335 ± 0.046	0.070 ± 0.032	0.039 ± 0.026
**DMS_{13}-PEG**	25	0.059 ± 0.037	0.030 ± 0.035	0.488 ± 0.059	0.014 ± 0.021	0.011 ± 0.010
**DMS_{30}-PEG**	25	0.000 ± 0.005	0.036 ± 0.046	0.237 ± 0.587	0.073 ± 0.084	0.006 ± 0.006

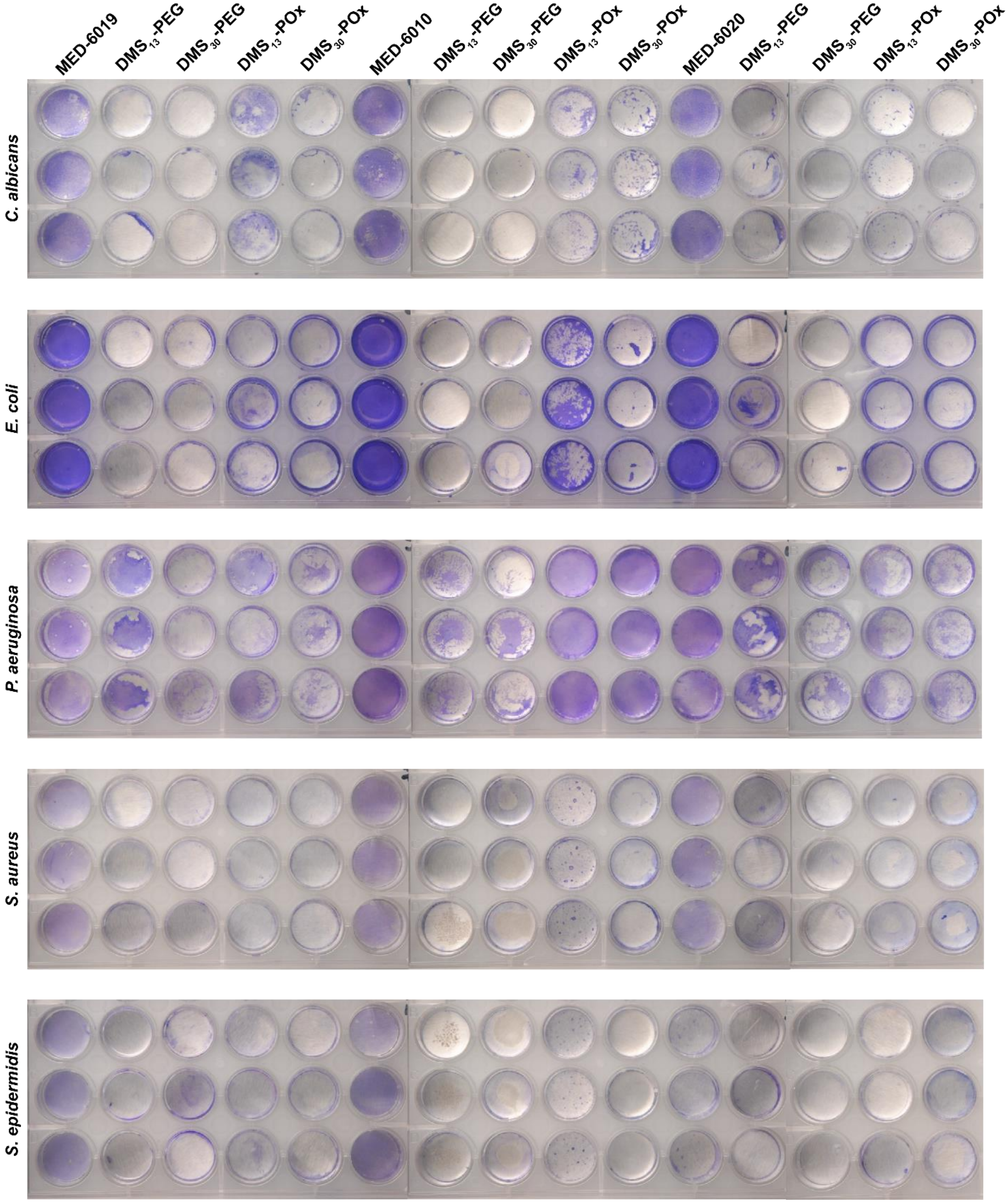

Figure S32. Images of unmodified silicones [MED-6019 (0 wt% Ph), MED-6010 (5.8 wt% Ph), and MED-6020 (8.5 wt% Ph)] and silicones modified with POx- and PEG-based SMAs after staining biofilm growth with crystal violet.

References

[1] A.C. Marmo, J.J. Rodriguez Cruz, J.H. Pickett, L.R. Lott, D.S. Theibert, H.L. Chandler, M.A. Grunlan, Amphiphilic silicones to mitigate lens epithelial cell growth on intraocular lenses, J. Mater. Chem B. 10(16) (2022) 3064-3072.
